# Alzheimer's Therapeutics Targeting Amyloid Beta 1–42 Oligomers I: Abeta 42 Oligomer Binding to Specific Neuronal Receptors Is Displaced by Drug Candidates That Improve Cognitive Deficits

**DOI:** 10.1371/journal.pone.0111898

**Published:** 2014-11-12

**Authors:** Nicholas J. Izzo, Agnes Staniszewski, Lillian To, Mauro Fa, Andrew F. Teich, Faisal Saeed, Harrison Wostein, Thomas Walko, Anisha Vaswani, Meghan Wardius, Zanobia Syed, Jessica Ravenscroft, Kelsie Mozzoni, Colleen Silky, Courtney Rehak, Raymond Yurko, Patricia Finn, Gary Look, Gilbert Rishton, Hank Safferstein, Miles Miller, Conrad Johanson, Edward Stopa, Manfred Windisch, Birgit Hutter-Paier, Mehrdad Shamloo, Ottavio Arancio, Harry LeVine, Susan M. Catalano

**Affiliations:** 1 Cognition Therapeutics Inc., Pittsburgh, Pennsylvania, United States of America; 2 Sanders-Brown Center on Aging, University of Kentucky, Lexington, Kentucky, United States of America; 3 Department of Pathology and Neurosurgery, The Warren Alpert Medical School of Brown University, Providence, Rhode Island, United States of America; 4 Stanford University Medical School Behavioral and Functional Neuroscience Laboratory, Palo Alto, California, United States of America; 5 NeuroScios, GmbH, Graz, Austria; 6 QPS Austria GmbH, Grambach, Austria; 7 Department of Pathology and Cell Biology and Taub Institute for Research on Alzheimer's Disease and the Aging Brain, Columbia University, New York, New York, United States of America; Huashan Hospital, Fudan University, China

## Abstract

Synaptic dysfunction and loss caused by age-dependent accumulation of synaptotoxic beta amyloid (Abeta) 1–42 oligomers is proposed to underlie cognitive decline in Alzheimer's disease (AD). Alterations in membrane trafficking induced by Abeta oligomers mediates reduction in neuronal surface receptor expression that is the basis for inhibition of electrophysiological measures of synaptic plasticity and thus learning and memory. We have utilized phenotypic screens in mature, *in vitro* cultures of rat brain cells to identify small molecules which block or prevent the binding and effects of Abeta oligomers. Synthetic Abeta oligomers bind saturably to a single site on neuronal synapses and induce deficits in membrane trafficking in neuronal cultures with an EC_50_ that corresponds to its binding affinity. The therapeutic lead compounds we have found are pharmacological antagonists of Abeta oligomers, reducing the binding of Abeta oligomers to neurons *in vitro*, preventing spine loss in neurons and preventing and treating oligomer-induced deficits in membrane trafficking. These molecules are highly brain penetrant and prevent and restore cognitive deficits in mouse models of Alzheimer's disease. Counter-screening these compounds against a broad panel of potential CNS targets revealed they are highly potent and specific ligands of the sigma-2/PGRMC1 receptor. Brain concentrations of the compounds corresponding to greater than 80% receptor occupancy at the sigma-2/PGRMC1 receptor restore cognitive function in transgenic hAPP Swe/Ldn mice. These studies demonstrate that synthetic and human-derived Abeta oligomers act as pharmacologically-behaved ligands at neuronal receptors - i.e. they exhibit saturable binding to a target, they exert a functional effect related to their binding and their displacement by small molecule antagonists blocks their functional effect. The first-in-class small molecule receptor antagonists described here restore memory to normal in multiple AD models and sustain improvement long-term, representing a novel mechanism of action for disease-modifying Alzheimer's therapeutics.

## Introduction

Disruption of the associative/dissociative balance in synaptic plasticity that is the basis of learning and memory begins in Mild Cognitive Impairment (MCI) and progresses as Alzheimer's disease continues. Evidence suggests this cognitive decline is caused by the accumulation of Abeta 1–42 oligomers in the brains of these patients [1]–[6]. Oligomers disrupt this balance by binding to plasma membrane proteins [7]–[15], changing intracellular calcium levels [11], [16]–[18], inducing tau mislocalization, disrupting microtubules [17], [18], altering membrane trafficking processes and surface expression levels of critical synaptic ion channels [19]–[21] and ultimately causing reversible spine loss in neurons [17], [22]–[25]. These disruptions result in reversible impairment of spatial memory [26], [27], that culminates in anterograde amnesia in the early stages of Alzheimer's disease [28]–[31].

Abeta 1–42 oligomer binding to some but not all neurons [19], and the reversibility of sublethal oligomer-induced cellular changes [17], [22] indicate that there may be a pharmacological basis for oligomer-induced synaptotoxicity, however, evidence that oligomers bind specifically and saturably to surface receptors has been underappreciated. While several cell surface proteins have been identified as receptors of Abeta oligomers [32], therapeutic ligands for these receptors have not been demonstrated to be effective in displacing bound Abeta oligomers. Additionally, there is disagreement over the identity of the form of Abeta oligomer responsible for human cognitive loss, creating difficulty in extrapolating *in vitro* results to *in vivo* efficacy.

In this study we utilized a phenotypic approach to discover small molecule drug candidates capable of blocking membrane trafficking dysfunction and synapse loss in mature primary hippocampal and cortical cultures caused by multiple forms of Abeta oligomers. This approach is capable of finding compounds which work by many different mechanisms, including direct disruption of Abeta oligomers; inhibition of Abeta oligomer binding; down-regulating expression of binding sites; or blocking signal transduction downstream from Abeta binding. We have found that the assays reliably identify compounds that inhibit Abeta oligomer binding and improve cognitive function in *in vivo* models of Alzheimer's disease. Active molecules discovered with this approach can be used to identify and characterize the receptors that mediate the binding and neuronal actions of Abeta oligomers. The behaviorally-effective compounds are potent and specific ligands for the sigma-2/PGRMC1 receptor. These findings support the idea that soluble Abeta oligomers act as pharmacological ligands on cellular receptors and can be antagonized with therapeutic small molecules.

## Materials and Methods

### Neuronal Cultures

All procedures were approved by the Institutional Animal Care and Use and Committee at Cognition Therapeutics and were in compliance with the Office of Laboratory Animal Welfare and the Guide for the Care and Use of Laboratory Animals, Eighth Edition.

Sprague-Dawley rats, 18 days pregnant, were euthanized by CO_2_ asphyxiation followed by cervical dislocation, and embryos were removed. Hippocampus and cortical tissue from the embryo brains were digested in 2.5% Trypsin (Life Technologies) to dissociate cells. Isolated cells were plated at a density of 4.6×10^4^ cells per cm^2^ in 384-well poly-D Lysine coated plates (Greiner) in Neurobasal Media (Life Technologies) supplemented with B27 (Life Technologies), Glutamax (Life Technologies) and antibiotics (penicillin, 50 units/ml and streptomycin 50 µg/ml, Life Technologies). Cultures were maintained at 37°C in 5% CO_2_ with weekly media change for 3 weeks prior to experimentation. These mixed cultures of hippocampal plus cortical neurons and glia were used for all of the *in vitro* experiments described.

### Trafficking Assay

Vesicular trafficking was measured using an adaptation of a method by Liu and Schubert [51]. Neurons were treated with compounds and/or Abeta oligomer preparations (0.086% DMSO in culture media) and incubated for 1 to 24 hr at 37°C in 5% CO_2_. Tetrazolium salts (3-(4,5-dimethylthiazol-2yl)-2,5diphenyl tetrazolium bromide, Roche Molecular Biochemicals) were added to a final concentration of 0.75 mM and incubated at 37°C for 60–90 min. Vesicular formazan remaining in cells was quantified by absorbance spectrometry (590 nm with 690 nm subtracted) following extraction with 1.6% Tween-20. All compounds were tested in quadruplicate wells for each concentration in at least 8 replicate experiments with data from all experiments pooled for analysis with means ± S.E.M.

### Oligomer Preparations

#### Synthetic peptide (high concentration)

Synthetic human Abeta 1–42 peptide (California Peptide Inc, catalog number 641-15; American Peptide Company, catalog number 62-0-80; or University of Pittsburgh Peptide Core facility, primary sequence DAEFRHDSGYEVHHQKLVFFAEDVGSNKGAIIGLMVGGVVIA) was treated according to published methods to remove any structural assemblies that may have formed during the synthesis, isolation and storage procedures [33], [34]. An Abeta monomer film was prepared by evaporating the 1,1,1,3,3,3,hexafluoro-2-propanol (HFIP) at room temperature from a solution of 0.253 mg Abeta 1–42 in HFIP at room temperature for 20 min using N_2_ gas. The film was then dissolved in dry DMSO (Sigma-Aldrich Catalog number D2650) and diluted to 100 µM with cold Basal Media Eagle media (BME, Life Technologies catalog 21010), followed by incubation at 4°C for 24 hr to form oligomers. The resulting oligomer preparations were centrifuged at 16,000×g to pellet any insoluble fibrils and the supernate was diluted in Neurobasal media prior to addition to cultures. All studies using synthetic oligomers were performed with this preparation unless otherwise specified. All lots of Abeta 1–42 are put through a strict quality control process before being used for experiments: A) vendor-provided MALDI-TOF spectra is checked for lack of truncated fragments; B) peptide content is >85%; C) preparation of oligomers at 100 µM as detailed above does not form a visible pellet of insoluble fibrils when centrifuged at 16,000×g; D) no cellular toxicity (as measured by fragmentation or loss of neuronal nuclei) is seen with 24 hr treatment of cells at concentrations up to 14 µM. For some experiments, peptide comprised of the same amino acid composition of Abeta 1–42 but in a randomized sequence was used as a control (“Scrambled Abeta 1–42”, American Peptide, Inc. 62-0-46B).

#### Synthetic preparation (low concentration)

Abeta 1–42 monomer film was formed from 20 µg synthetic Abeta 1–42 in HFIP. The film was dissolved in 200 µl DMSO, mixed with 10 ml PBS and incubated for two hours at room temperature at a final concentration of 500 nM to form the Abeta 1–42 oligomers. Oligomerization was stopped by adding 100 mg BSA and the sample was loaded onto a 250 ml column of Sephadex G-75 equilibrated with MEM containing 2 mg/ml BSA. The column was eluted with MEM + BSA and the void volume containing high molecular weight oligomers was collected. Pooled fractions of interest were concentrated with 100 kDa filters (Amicon Ultra). The retentate was diluted in PBS and concentrated to a small volume then rediluted with PBS and re-concentrated using 100 kDa filters. This dilution/re-concentration process was repeated two additional times to remove residual BSA.

### Semi-synthetic oligomers

Whole rat brain was homogenized 1∶7 (w/v) in 20 mM Tris-HCl, 137 mM NaCl, pH 7.6 with 1 mM EDTA and 1 mg/ml protease inhibitor cocktail (Sigma P8340) and centrifuged at 105,000×g for 1 hour at 4°C. Supernates were immunodepleted using protein-A and protein-G Sepharose (Pierce). Filtrates were concentrated thirty-fold using Amicon 3 kDa filters. After dilution to 500 uL in TRIS-buffered saline, Abeta 1–42 monomers were added to this size excluded homogenate to a final concentration of 50 µM and incubated overnight at 4°C. Abeta oligomers were immunoprecipitated with 6E10-conjugated agarose columns overnight at 4°C, washed 3 times with PBS (Pierce) and eluted using Gentle AG/Ab Elution Buffer (Pierce).

### AD patient-derived Abeta

Human AD patient (Braak V/VI) and age-matched control hippocampal brain specimens with <24 hour post-mortem interval were obtained from the Brown Brain Bank. Human brain samples were obtained at autopsy from cognitively normal age-matched control and Alzheimer's disease (AD) patients. In all cases, consent for possible use in research was obtained from the next of kin in accordance with protocols approved by the Lifespan/Rhode Island Hospital Institutional Review Board for human studies IRB #0083-03. All samples were numbered to remove identifying personal information prior to their investigational use.

Human brain tissue was homogenized (Dounce glass homogenizer) 1∶7 (w/v) in ice cold buffer (20 mM Tris, 137 mM NaCl, pH 7.6 with 1 mM EDTA and 1 mg/mL protease inhibitor cocktail Sigma P8340) and then centrifuged at 105,000×g for 1 hour at 4°C. Supernates were then immuno-depleted with protein-A and protein-G Sepharose (Pierce) and then centrifuged in Amicon Ultra 100 kDa filters at 14,000 g for 10 minutes at 4°C. The eluted samples were then concentrated approximately 30-fold using Amicon 10 kDa filters to obtain the10–100 kDa size-fractionated oligomers which were then captured on 6E10-conjugated agarose columns (Pierce NHS-Activated Dry Agarose Resin, catalog # 26196). Immunoisolate material was released from the 6E10-agarose using gentle elution buffers (Pierce) and then desalted by repeated washes with PBS in Amicon 10 kDa MW filters followed by storage in aliquots at −80°C until further use. Use of brain specimens taken after autopsy meet the criteria for exemption (Exemption 4) from the requirements in DHHS regulations (45 CFR 46).

#### Abeta binding Assay

To assess the ability of test compounds to prevent the binding of Abeta oligomers, cultures were treated with compounds for 30 minutes, followed by synthetic Abeta 1–42 oligomer preparation treatment for 60 min (total Abeta concentration 0.5 µM, equivalent to Kd concentration). Alternatively, displacement of prebound oligomers was evaluated by adding oligomers 60 min prior to the addition of compounds, followed by additional 30 minutes incubation. Cells were fixed with 3.75% formaldehyde for 15 min, blocked with 5% normal goat serum and 0.5% Triton X-100 and incubated with primary antibodies for Abeta (1 µg/ml 6E10 or 4G8, Covance catalog numbers SIG-39320 and SIG-39330, respectively), MAP-2 (0.2 µg/ml Chemicon), Synaptophysin-1 (1 µg/ml, Anaspec), glial fibrillary acidic protein (GFAP, 1 µg/ml, Thermo-Fisher) and fluorescently labeled secondary antibodies (2 µg/ml, Invitrogen). Images were acquired on a Cellomics VTi automated microscope with a 20X, 0.75 NA objective and analyzed using ThermoFisher/Cellomics Neuronal Profiling bioapplication set to measure punctate labeling of Abeta and synaptophysin-1 along MAP-2 labeled neurites. For each replicate experiment, at least 100 neurons were sampled from 4 replicate wells for each experimental condition (400 to 500 neurons per experimental condition). The number of replicate experiments is reported for each experiment. All data presented for Abeta binding to neurons represents total intensity of Abeta label in neurite spots per neuron, in relative fluorescent units (RFU), unless otherwise indicated.

Measurements of Abeta binding to glia were made with a separate algorithm (ThermoFisher/Cellomics Target Activation bioapplication) which identifies cells lacking MAP2 labeling. Glial cells were analyzed after separating them based on their nuclear morphology as determined by the characteristics of their DAPI labeling, as described in **Fig. S1**. All data for Abeta binding to glia represent total intensity of the Abeta label in the cell body in relative fluorescent units (RFU), unless otherwise indicated.

### Synapse counting assay

Cultures were treated with compounds for 60 minutes prior to addition of 6 µM (maximum from trafficking dose-response curve) of synthetic Abeta 1–42 oligomers and incubated for 24 hrs. Cultures were fixed and immunofluorescently labeled and imaged as for the Abeta binding assay. The ThermoFisher/Cellomics Neuronal Profiling bioapplication was used to count synaptophysin-immunopositive puncta along MAP2-labeled neurites. The number of puncta per unit length of neurites was used to determine effects of treatments. To attain statistical power of 80% and a p value of less than 0.05 (determined using G*Power software [35]), twenty replicate wells were treated per experiment plate and compared to control wells on the same plate and experiments were repeated four times.

### Behavioral Studies

All procedures were approved by the Institutional Animal Care and Use and Committee at Columbia University and Stanford University and were in compliance with the Office of Laboratory Animal Welfare and the Guide for the Care and Use of Laboratory Animals, Eighth Edition. Behavioral testing of wild-type, 3–4 month old, male, C57/BL6 mice, injected bilaterally into their hippocampus with Abeta oligomers and testing in the fear conditioning task was done according to published methods [36]. Animals were anesthetized with 20 mg/kg Avertin prior to implantation of cannulas into the hippocampus. Testing of transgenic hAPP Swedish/London mice in the Morris water maze or fear conditioning tasks was done according to published methods [37], [38]. To measure brain concentration of drugs, brains were homogenized in 1 ml PBS per gram of brain tissue using a handheld homogenizer. Brain homogenate was then extracted with three volumes of ice cold methanol on ice for 15 minutes and centrifuged for 15 min. The supernates were analyzed by LC/MS/MS. Samples were compared to a calibration curve prepared in a similar manner by spiking blank control homogenate with standards prepared in DMSO and then extracted as above. At the end of all behavioral testing, animals were euthanized with CO_2_ followed by cervical dislocation.

### Western Blot Analysis of Abeta Oligomers

Since SDS-containing denaturing gels have been shown to create artifactual assembly and disassembly products of Abeta oligomers, non-denaturing conditions were used to characterize Abeta preparations. Abeta oligomer preparations were run on 4–15% Tris-HCl nondenaturing gels (BioRad), transferred to nitrocellulose, probed with 6E10 mouse monoclonal antibody to Abeta (Covance catalog number SIG-39320) followed by secondary goat anti-mouse horse radish peroxidase-conjugated secondary antibody (Millipore catalog number AP308P) and visualized with chemiluminescent detection (Immun-Star, BioRad).

### Abeta ELISA

Determination of oligomeric and monomeric concentrations of Abeta contained in a given Abeta preparation was done according to the method of LeVine [39]. Briefly, total Abeta concentration is measured by two-site ELISA using different capture (6E10 monoclonal antibody, Covance SIG-39320) and detection (4G8- conjugated to biotin, Covance, SIG39240) antibodies and quantifyied with streptavidin-horseradish peroxidase (Millipore catalog number AP308P). Oligomerized Abeta is measured in the same samples by a single-site ELISA in which monoclonal antibodies to the same epitope are used for capture (4G8, Covance SIG-39330) and detection (4G8-biotin). The 4G8-biotin cannot bind to captured monomer because the epitope is blocked by the capture antibody, so only oligomers are detected with this configuration. The streptavidin-horseradish peroxidase therefore reports only oligomeric Abeta which has additional exposed 4G8 epitopes. Monomer concentration is calculated as the difference between total Abeta concentration and oligomer concentration.

### Sigma-2 radioligand binding

Radioligand competition assays were performed in membranes from human Jurkat cells using 5 nM [^3^H]1,3-di(2-tolyl) guanidine in the presence of 1 µM (+)-pentazocine with 10 µM haloperidol to define non-specific binding [40].

### Statistical Analysis

For all experiments involving quantification of Abeta immunofluorescent intensity, at least four replicate, multiwell plates were analyzed, with a minimum of 4 replicate wells per condition on each plate and 16 fields imaged per well. Averages of total puncta intensity per neuron (approx. 90 neurons per well) were calculated for each well analyzed. These well averages were tested for normality using a KS distance test before being analyzed for treatment differences using ANOVA and Bonferroni's multiple comparison post-test or pairwise Student's t-test as indicated (Graphpad Prism).

### Animal Welfare

These studies were carried out in strict accordance with the recommendations in the Guide for the Care and Use of Laboratory Animals of the National Institutes of Health. The protocol was approved by the Institutional Animal Use and Care Committees at Cognition Therapeutics, Columbia University, Stanford University and QPS Austria GmbH.

## Results

### *In vitro* assay system: Mature (21DIV) primary hippocampal and cortical cultures in 384- well microtiter plates

**Figure 1:**
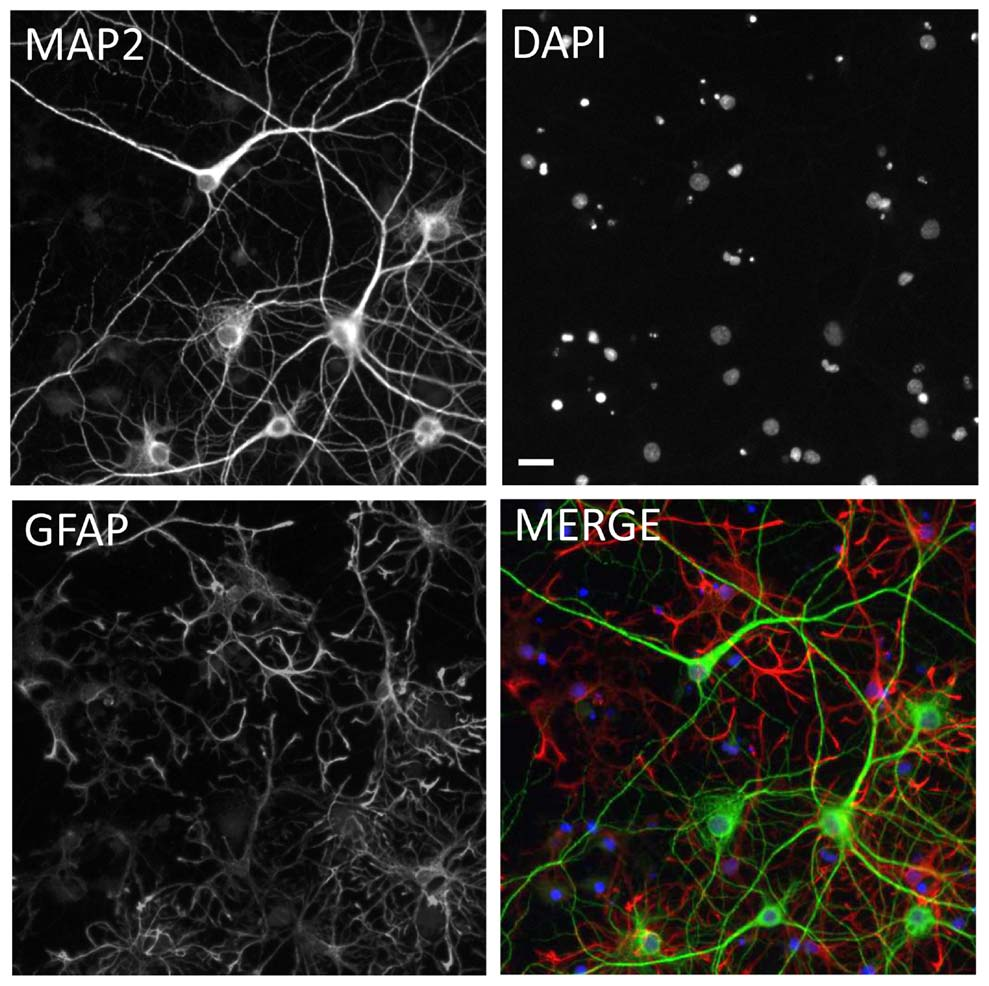
Immunofluorescent labeling of mixed hippocampal/cortical cultures. **A**, MAP2 labeled neurons. **B**, DAPI-labeled nuclei. **C**, GFAP labeled glia and Nuclei. **D**, Merged composite of all three images. Based on untreated control wells from 104 experimental plates, the percentage of neurons in the cultures was 26.0±1.1% (Mean ± S.E.M.). Scale bar  = 20 microns.

We chose to use primary rat neurons grown for at least 21 days *in vitro* (21DIV) as a basis for our assays. These neurons express the full complement of synaptic proteins characteristic of neurons in the mature brain, and exhibit oscillatory electrical activity *in vitro* [41]. Neurons and glia in primary culture have molecular signaling networks exhibiting excellent registration with intact brain circuitry, and for this reason have been used for over two decades as a model system for learning and memory [42], [43]. More complex systems such as acute or organotypic brain slices are very useful but not amenable to high-throughput screening. Neuronal cell lines can be used in high-throughput screens, but they do not replicate the electrophysiological state-dependent signaling of primary neuronal cultures and are unlikely to adequately model the subtle alterations in this signaling that are caused by oligomers during the earliest manifestations of the disease state [44]. For this reason, primary neuronal cultures are an acceptable compromise between throughput and fidelity and form the basis for our assays. These cultures are a mixture of both MAP2-positive neurons and GFAP-positive glia as characterized with immunofluorescent labeling (**Fig. 1**) with 26.0±1.1% (Mean ± S.E.M., N = 104 experimental plates) of the cells being neurons.

### Characterization of Abeta oligomer preparations

The Abeta peptide exists in a variety of structural forms ranging from monomer, to various shapes and sizes of oligomers, to infinite polymers known as fibrils. The equilibrium between these different assembly forms in the human brain and how this equilibrium is affected in the disease state is currently unknown. Evidence suggests that threshold concentrations of the water soluble oligomeric forms of Abeta are the most toxic, and correlate with disease state [45]–[47]. Oligomers of various sizes can be seen in varying amounts when synthetic monomeric Abeta peptide solutions are allowed to oligomerize, or when Abeta is immunologically isolated from human or transgenic animal brain, with partial overlap of oligomer sizes in these different preparations [45], [48]. When they have been compared side-by-side in the same assay [26], all of these oligomers are neuroactive, but have different potencies. We took a similar comparative approach and looked for small molecules that were active at stopping the downstream toxicities of several Abeta oligomer preparations *in vitro*. This allowed us to distinguish common pharmacophores that yielded nodal points of intervention in the signaling cascade.

**Figure 2:**
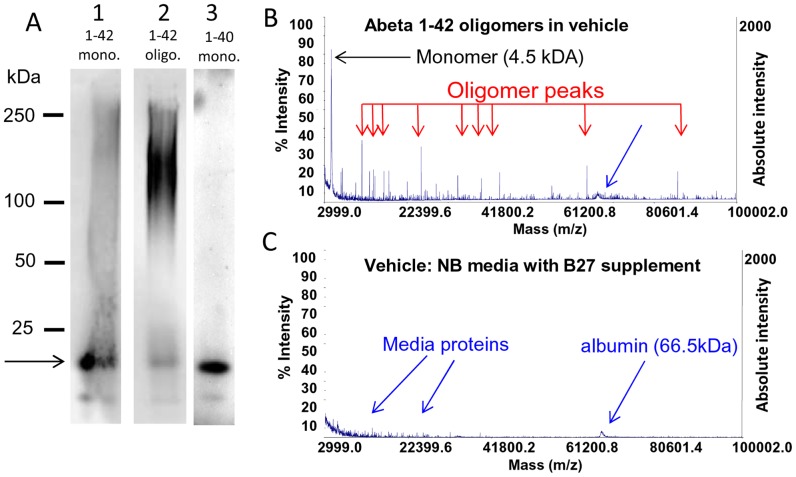
Characterization of synthetic human Abeta 1–42 oligomers by non-denaturing Western blot, MALDI-TOF. **A**, Freshly prepared solutions of synthetic human Abeta 1–42 (lane 1) or 1–40 (lane 3) peptide loaded onto non-denaturing western gels immediately after reconstitution contain large amounts of monomer (arrow; fainter lower molecular weight band represents peptide degradation product) and little higher molecular weight material. In contrast, the same solution of Abeta 1–42 peptide that is allowed to oligomerize for 24 hours (lane 2) contains much larger amounts of higher molecular weight material >50 kDa, and less monomeric protein. The full length of gel lanes are shown from loading well to dye front. Note that oligomers run differently on non-denaturing gels than globular molecular weight protein size standards [49]. **B**. The presence of significant amounts of monomer in oligomer preparations is also confirmed by MALDI-TOF analysis of the same Abeta 1–42 oligomer preparation that shows both a 4.5 kDa monomer peak and multiple lower abundance peaks corresponding to oligomers of various sizes. MALDI-TOF (detection range 3–100 kDa) of vehicle (media without Abeta) is shown below for comparison (**C**).

Synthetic preparations of Abeta oligomers were analyzed on non-denaturing western gels so that oligomeric forms of Abeta would not be disrupted. Under these conditions oligomers run differently on these gels than globular molecular weight protein size standards [49]. Accordingly, we used several methods to determine the relative concentrations and apparent molecular weight size ranges of monomer and oligomer in a given Abeta oligomer preparation. Oligomer preparations made overnight from Abeta 1–42 run as a broad band between 75 and 150 kDa and contain some monomer which runs at 15 kDa (**Fig. 2A** **, lane 2**). Fresh preparations of Abeta 1–42 (treated with HFIP to remove prior structural assemblies), added immediately to the gel run mostly as monomer but some high molecular weight species still form quickly (**Fig. 2A** **, lane 1**). Fresh preparations of Abeta 1–40 run as monomer with an apparent molecular weight of 15 kDa with no higher molecular weight assemblies (**Fig. 2A** **, lane 3**). The presence of significant monomer in oligomer preparations is also confirmed by MALDI-TOF analysis of oligomer preparation that shows both a 4.5 kDa monomer peak and multiple peaks corresponding to oligomers (**Fig. 2B**) while MALDI-TOF analysis of vehicle without Abeta show only small peaks corresponding to media proteins (**Fig. 2C**, [50]). Preparations of oligomeric Abeta and fresh monomeric Abeta were analyzed using two-site binding ELISAs which measure total peptide and single-site ELISAs which determine oligomeric Abeta and allow the total amount of monomeric Abeta to be estimated [4], [5], [39]. This analysis shows that the unfractionated oligomer preparations used typically contain about 1/3 monomer and that fresh monomer preps quickly form measurable amounts of oligomer (**Table 1**).

**Table 1:** Concentration of oligomer and monomer in Abeta preparations as determined by ELISA.

ELISA	Alzheimer's patient-derived Abeta	Semi-synthetic Oligomers	Synthetic Oligomers (low concentration)	Synthetic Oligomers (high concentration)	Fresh Monomer
Oligomer conc.	137 pM	90.5 nM	65.5 nM	1.5 µM	170 nM
Monomer conc.	0	44.8 nM	0	0.7 µM	2.83 µM

**Figure 3:**
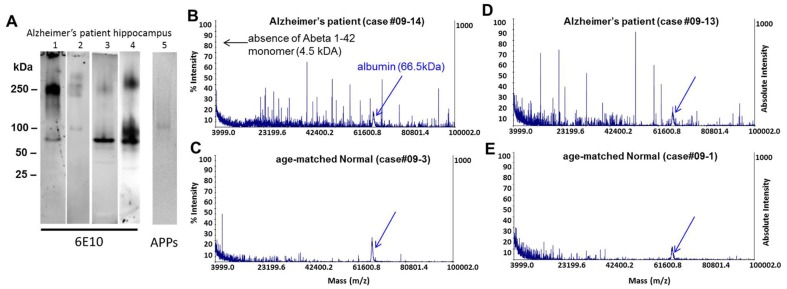
Characterization of human Abeta 1–42 oligomers isolated from patient frozen, unfixed 1 gram brain samples by non-denaturing Western blot, MALDI-TOF and ELISA. **A**, Non-denaturing Western blots of immunoprecipitated Alzheimer's patient hippocampal samples demonstrates heterogeneous populations of oligomer assemblies. 6E10 antibody labeling of western blots from four different AD patients (lanes 1–4) detects major bands ≥250 kDa, and multiple discrete bands between 50–75 kDa. In contrast, APP antibody detects a faint band at 125 kDa (lane 5). Significant amounts of monomeric Abeta 1–42 were not observed in any individual. MALDI-TOF analysis of immune-precipitated human brain samples demonstrates heterogeneous populations of oligomer assemblies, both between individual Alzheimer's patients (**B, D**) and between age-matched histologically normal individuals (**C, E**). Significant amounts of monomeric Abeta 1–42 were not observed in any individual. Albumin was added to samples as an internal size control (arrow in **B–E**).

Quantification of relative concentrations of oligomeric and monomeric peptide in a freshly prepared solution of Abeta 1–42 (corresponding to lane 1 in **Fig. 2A**, 3 µM total Abeta concentration), Abeta 1–42 oligomers (corresponding to lane 2 in **Fig. 2A**, 3 µM total Abeta concentration) and Alzheimer's patient-derived Abeta (**Fig. 3**) measured by two-site (total) and single-site (oligomer) ELISA [4], [5], [39]. Both synthetic peptide solutions contain monomer. In contrast, Abeta peptide immunopurified from a 1 gram tissue sample of human Alzheimer's patient hippocampus contains no detectable monomer.

Non-denaturing Western blots of immunoprecipitated samples from 1 gram weight frozen unfixed post mortem hippocampus from Alzheimer's patients demonstrated heterogeneous populations of oligomer assemblies (**Fig. 3A**). Labeling of western blots from four different AD patients (lanes 1–4) with 6E10 antibody detects major bands greater than or equal to 250 kDa, and multiple discrete bands between 50–75 kDa. In contrast, APP antibody detects a faint band at 125 kDa (lane 5). Significant amounts of monomeric Abeta 1–42 were not observed in any individual. MALDI-TOF analysis of immunoprecipitated human brain samples demonstrates heterogeneous populations of oligomer assemblies, both between individual Alzheimer's patients (**Fig. 3B, D**) and between age-matched histologically normal individuals (**Fig. 3C, E**). Significant amounts of monomeric Abeta 1–42 were not observed in any individual. Thus our characterization of Alzheimer's patient brain-derived Abeta supports the observation that there is overlap in the molecular weight range in Abeta species seen in synthetic and human-derived oligomer preps, so that synthetic preps model some aspects of the human oligomers. It also demonstrates that synthetic Abeta preparations are a mixture of monomer and oligomeric forms of the protein; the relative concentration of each depends on the amount of time it is allowed to assemble.

### Characterization of trafficking assay

Since synaptic and memory deficits, rather than widespread cell death, predominate at the earliest stages of Alzheimer's disease, assays that measure these changes are particularly well suited to discovering small molecule inhibitors of oligomer activity. The MTT assay is frequently used as a measure of toxicity in cultured cells, yet we and others have reported that it can also be used to measure membrane trafficking changes [51]–[53]. Yellow tetrazolium salts are endocytosed by cells and reduced to insoluble purple formazan in the endosomal pathway. The level of purple formazan is a reflection of the number of actively metabolizing cells in culture, and reduction in the amount of formazan is taken as a measure of cell death or metabolic toxicity in culture.

**Figure 4:**
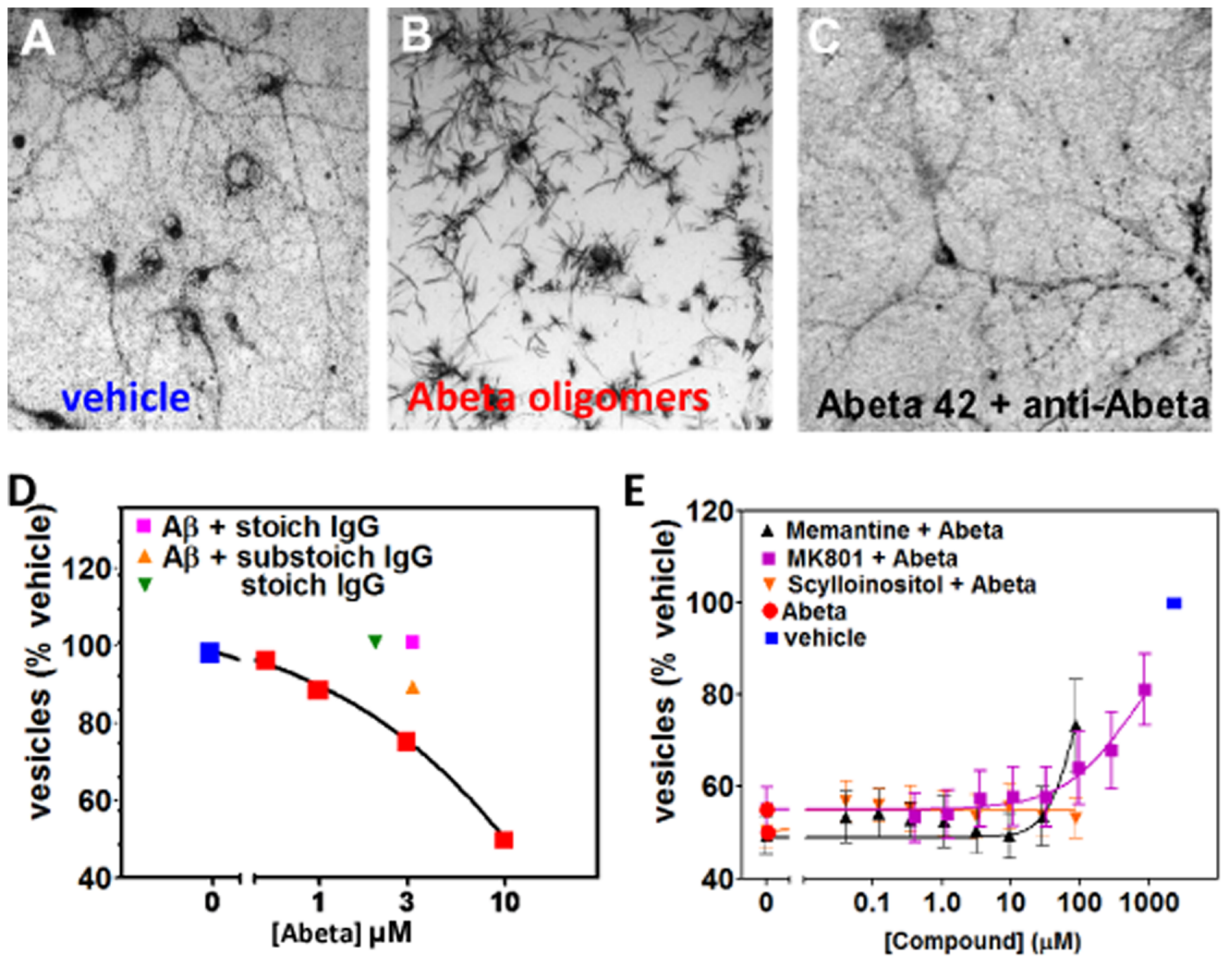
Characterization of Membrane Trafficking Assay. Membrane trfficking rate is measured by quantifying the number of intracellular vesicles labeled with endocytic cargo dye in mature primary hippocampal cultures (≥21 DIV). Over time, the dye-filled vesicles (**A**) are trafficked out of cells via exocytosis, and the amount of dye in vesicles decreases as it crystalizes on the cell surface as needle-shaped crystals (**B**). Abeta oligomer treatment affects the rate of membrane trafficking; 60 minutes following addition of MTT reagent, when vehicle treated neurons (**A**) still contain labeled vesicles, Abeta oligomer-treated neurons (**B**) have already exocytosed labeled vesicles. **C**. Addition of antibody to Abeta (6E10) to cultures prevents Abeta oligomers from affecting trafficking rates. Amount of labeled vesicles is quantified (y-axis in **D**) as a percentage of vehicle-treated values. **D**. Concentration-dependent effect of synthetic Abeta oligomer preparation on vesicular labeling relative to vehicle treated cells is blocked by antibody IgG to Abeta. **E**. Memantine (MEM), MK-801, and scyllo-inositol were run as controls in the membrane trafficking assay.

We have adapted the exocytosis assay for use with mature primary neuronal cultures grown for 3 weeks *in vitro*. When cells are observed through a microscope approximately 60 min following the addition of tetrazolium salt, vehicle-treated cells appear filled with purple formazan-containing vesicles (**Fig. 4A**), while cells treated with Abeta oligomers have trafficked these vesicles to the plasma membrane surface and released their contents at the cell surface during exocytosis, leading to precipitation of the water-insoluble dye at the cell membrane (needle-shaped crystals, **Fig. 4B**). This decrease can be blocked by adding stoichiometric amounts of anti-Abeta monoclonal antibody 6E10 to the cultures prior to oligomer addition (**Fig. 4C, D**) while antibody alone has no effect on the neurons at low concentrations. Abeta oligomers accelerate the process of exocytosis, causing a dose-dependent decrease in the amount of intracellular vesicles (puncta) filled with reduced purple formazan compared to vehicle-treated cultures (**Fig. 4D**, [51]). Under these circumstances, there is no overall change in the total amount of reduced formazan, simply a shift in its subcellular location and morphology. We tested several compounds that have, been reported to block the effects of Abeta oligomers, including the sugar alcohol scyllo-inositol (AZD-103), the nAChR antagonist hexamethonium bromide, and the NMDAR antagonists MK-801 and memantine [21], [23], [54]; only memantine was active, weakly blocking oligomer effects at very high, non-physiological doses above 100 µM (**Fig. 4E**, [55]).

We have optimized this assay for performance in 384-well microtiter plates with automated liquid handling robotics for compound formatting and assay plate stamping. The amount of labeled vesicles in treated cells is quantified by automated image processing or by spectrophotometry following extraction from cells and is calculated as a percentage of vehicle-treated values. Control wells consist of Abeta and vehicle treatment without test compounds. We set a passing window for controls (Abeta/vehicle) of between 50% and 80% with greater than 85% of test runs passing this criteria, with a resulting z' score of 0.285 [56]. In the prevention configuration of this assay, compounds are added to neuronal cultures first, followed by oligomers. Compounds are also tested in the treatment configuration of this assay in which oligomers are added to the cultures first, followed by compound addition. Compounds active in both assay configurations are more likely to be intervening at pharmacologically tractable points in the signaling cascade. Hit compounds that are effective at blocking Abeta oligomer-induced deficits but do not affect normal trafficking in the absence of Abeta were forwarded to subsequent screens.

**Figure 5:**
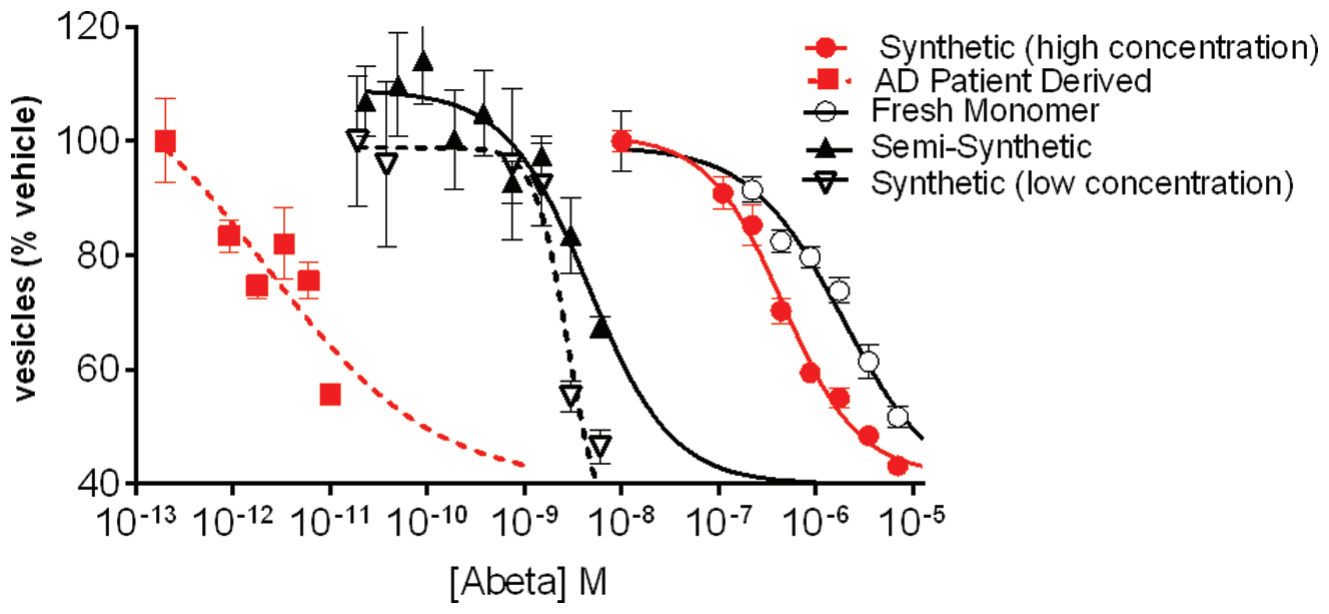
Relative potency of Abeta preparations in membrane trafficking assay. Synthetic human Abeta 1–42 oligomer (high concentration), freshly made monomer, synthetic oligomers (low concentration), semi-synthetic oligomers and human Alzheimer's patient derived oligomers were dosed in the membrane trafficking assay. All Abeta preparations alter membrane trafficking rates but with different EC_50_ concentrations and different exposure times to B_max_, similar to literature reports (**Table 2**).

The potency of Alzheimer's patient-derived Abeta, synthetic Abeta oligomer preparations (high concentration oligomers formed at 100 µM and low concentration oligomers formed at 500 nM) and monomer dosed in the membrane trafficking assay is shown in **Fig. 5** (all concentrations expressed as total Abeta concentration). The duration of treatment required to achieve maximal effects on membrane trafficking as well as the EC_50_ concentrations of each Abeta preparation vary widely, with Alzheimer's patient-derived Abeta being several orders of magnitude more potent than synthetic Abeta oligomers (**Fig. 5**, **Table 2**). Fresh monomer is the least potent of all preparations, requiring 24 hours treatment to have any effect on trafficking. The relatively low potency of synthetic high concentration oligomer preparations may be partially due to the significant concentrations of Abeta monomers in these preparations. We have found that removing monomer from our synthetic prep using size exclusion chromatography (e.g. the synthetic low concentration oligomer preps, **Fig. 5**) yields a preparation that is of intermediate potency between synthetic high concentration oligomer preps and human AD patient-derived preparations (**Fig. 5**). Synthetic high concentration Abeta oligomer preparations contain significant amounts of monomer when analyzed via western blot (**Fig. 2A**) or quantified via two-site and single-site binding ELISAs (**Table 1** and references [5], [39]), whereas human AD patient-derived Abeta does not. Fresh monomer added back into synthetic low concentration oligomer preparations has been shown to lower oligomer-induced membrane trafficking deficits [57]. This makes the testing of fresh monomeric control conditions critical for proper interpretation of results obtained with any synthetic oligomer preparations.

**Table 2:** EC_50_ and time to maximum effect of Abeta preparations in membrane trafficking assay.

Membrane Trafficking Assay	Alzheimer's patient-derived Abeta	semi-synthetic oligomers	Synthetic oligomers (low concentration)	synthetic oligomers (high concentration)	Fresh monomer
EC_50_	3.5 pM	5.9 nM	2.7 nM	460 nM	9600 nM
Treatment duration for maximum response	1 hr	1 hr	1 hr	24 hr	24 hr

EC_50_ for Alzheimer's patient derived Abeta, semi-synthetic oligomer, and high concentration synthetic oligomer based on ELISA measurement of Abeta oligomers in the preparation. EC_50_ for synthetic oligomer and fresh monomer determined according to total Abeta added to the assay.

### Characterization of Abeta oligomer binding to neurons and glia

**Figure 6:**
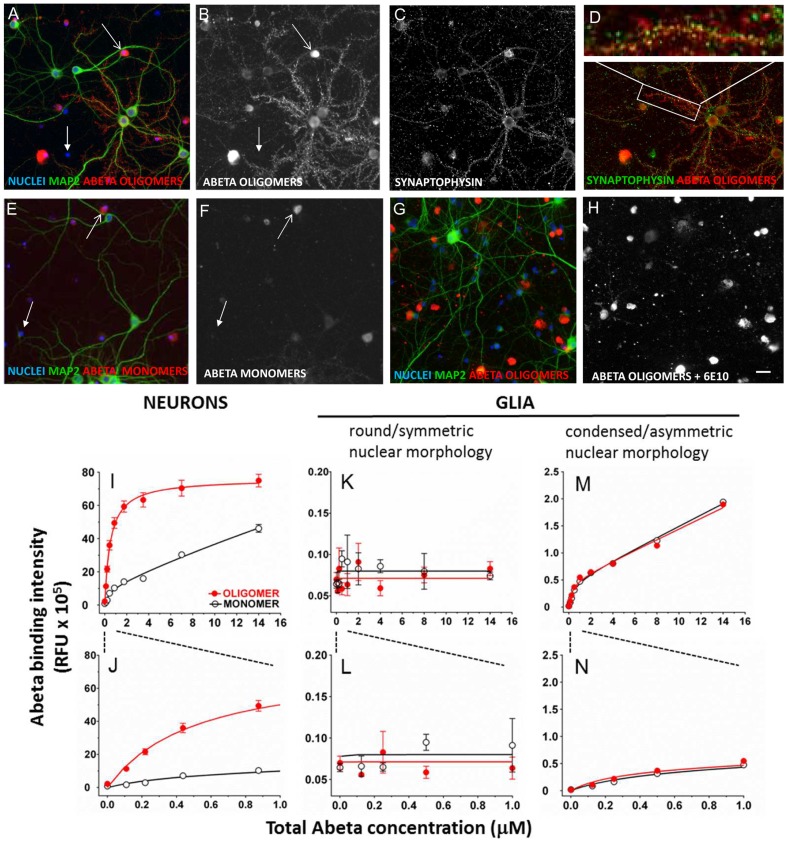
Abeta oligomers bind to a single saturable receptor site on neuronal synaptic puncta. **A, B**. Abeta 1–42 oligomers bind to some but not all neurons when added to cultures for 60 minutes (total Abeta concentration  = 440 nM, visualized with 6E10 immunolabeling). A subset of neurons (immunopositive for MAP2, green) exhibit punctate postsynaptic oligomer binding (red) along their neurites; 37%±3% of these puncta colocalize with presynaptic terminals immunolabeled for synaptophysin (**C, D**). Nuclei (DAPI +, blue) of several non-neuronal cells (glia, MAP2-negative) exhibit Abeta binding to the cell body. **B, F, H**. Immunolabeling of Abeta, alone, is shown for clarity. **E, F**. Fresh Abeta 1–42 monomers, added to cells with identical concentrations and conditions, is characterized by very low intensity punctate labeling on neurites and labeling of neuron and glia cell bodies. **G, H**. 6E10 (monoclonal antibody to Abeta 3–8) added to cultures prior to oligomers blocks binding of Abeta oligomers to neurite puncta but not to cell bodies of neurons or glia. **I**. Binding isotherms for Abeta oligomers (red closed circles) and fresh Abeta monomer (black open circles; treatment for 60 minutes with 44 nM- 14 µM total Abeta concentration) indicate that oligomer binding to neuronal puncta fits a single-site, saturable model (Kd = 518±41 nM, **Table 3**). Binding of fresh monomer fits a two site model with a high affinity site (412±48 nM) and a second non-saturable binding site. **J**. Same data as **I**, expanded to show concentrations used for competition binding studies (440 nM total Abeta concentration); at these low concentrations binding intensity of oligomers to neuronal synaptic puncta is five-fold higher than with monomer. **K, L**. Binding of synthetic Abeta oligomers and fresh monomer to glial cells with round/symmetric nuclear morphology (closed arrowheads in **B**, **F**) is not above background levels (zero Abeta concentration in all graphs). **M, N**. Binding of synthetic Abeta oligomers and fresh monomer to glia with condensed/asymmetric nuclear morphology(open arrowheads in **B**, **F**) fits a two site model with a high affinity site (Kd = 289±150 nM) and a second non-saturating site (Kd>1 M). Scale bars  = 20 µm.

We utilized a high content multiparameter automated imaging assay for measuring both Abeta binding and synapse loss via immunofluorescence. We characterized the binding of Abeta oligomers and monomers to neurons and glia *in vitro* by treating cultures with Abeta preparations for 1 hour, followed by fixation and processing for 4G8 immunolocalization. Synthetic Abeta oligomers exhibit binding to specific postsynaptic sites present on 30–50% of hippocampal neurons in primary cultures (**Fig. 6A–D**) as has been reported in the literature [19], [58]. This *in vitro* observation is similar to the percentage of neurons reported to be labeled with Abeta oligomers in human AD patient brain [58]. Oligomers also bind to cell bodies of neurons and glia. Freshly prepared monomeric Abeta binds much less intensely than oligomers to the same cellular elements (**Fig. 6E, F**). Co-treatment of Abeta oligomers with an N-terminal monoclonal antibody to Abeta (6E10, 26 µg/ml) completely eliminates binding to synaptic puncta, but cell body labeling of neurons and glia was still observed (**Fig. 6G, H**).

#### Neurons

The binding of synthetic Abeta oligomers to puncta on neurons is saturable and fits a single site binding model with a Kd of 512±41 nM (mean ± S.E.M., **Fig. 6I**, with expanded x-axis to show lower concentrations used in competition experiments in **Fig. 6J**), similar to literature values [9]. In contrast to oligomers, equal concentrations of fresh monomer results in less intense punctate binding than that of oligomers (**Fig. 6I, J**) and fits a two site binding model, with a high affinity site (Kd = 412±480 nM) and a low affinity site which is effectively non-saturable (Kd>1 mM). The high affinity Kd values for monomer and oligomer binding are not significantly different from each other, but the monomer binding intensity is 60–70% lower at each concentration. Whether this high affinity binding actually represents monomer or small amounts of oligomer that form during the course of the one hour treatment is not clear; rapid formation of small amounts of oligomer even in freshly prepared monomer is apparent from our oligomer-specific ELISA and Western Blots (**Table 1**, **Fig. 2**). The non-saturable monomer binding may represent fibrils formed from high concentration monomer nucleated by binding to negatively charged surface molecules located on the cell body, and subsequent polymerization [59].

#### Glia

We rarely observe punctate oligomer binding to glial cell processes *in vitro*, whereas glial cell body labeling is common. The intensity of glial cell body Abeta labeling is bimodal, and correlates dramatically with nuclear morphology. We observe two broad categories of DAPI-labeled nuclear morphology in MAP2-negative glial cells *in vitro*: round/symmetric nuclei and condensed/asymmetric nuclei (see **Fig. S1**; this difference may result from the serum-free media conditions. Additional characterization of these different glial populations requires further study). Binding of Abeta oligomers (**Fig. 6B**, closed arrow) or fresh monomer (**Fig. 6F**, closed arrow) to cell bodies of glia with round/symmetric nuclear morphology is not significantly above background at any concentration. Bright binding of Abeta oligomers and monomer to glial cell bodies with condensed/asymmetric nuclei is more than 10 times as intense, (open arrows in **Fig. 6B, F**) and both monomer and oligomer binding to these cell bodies fits a 2 site binding model with a high affinity site (oligomer Kd = 289±150 nM, monomer Kd = 445±221 nM) and a second non-saturable site. (**Fig. 6M**, N). The high affinity Kd values for monomer and oligomer binding and the intensity of binding are not significantly different. This suggests that either 1) oligomer does not bind to glial cell bodies with asymmetric/condensed nuclear morphology, or 2) oligomer receptor density is so low that even the small amount of monomer that oligomerizes under experimental conditions is able to occupy 100% of available receptor sites. As with neuronal synaptic puncta, non-saturable binding to these glial cell bodies may represent polymerization of monomeric Abeta into fibrils. Binding affinities for oligomers and monomers to each cell population is summarized in **Table** **3**.

**Table 3:** Binding affinity (Kd) of Abeta preparations to cellular compartments of neurons and glia.

Cell Type	NEURONS	GLIA	
Compartment	synaptic puncta	cell bodies with condensed/asymmetric nuclear morphology	cell bodies with round/symmetric nuclear morphology
Oligomer	Site 1: 518±41 nM	Site 1: 289±150 nM	NS
		Site 2: >1 mM	
Fresh	Site 1: 412±480 nM	Site 1: 445±211 nM	NS
Monomer	Site 2: >1 mM	Site 2: >1 mM	

Mean Kd ± S.E.M. Data are results of 13 replicates for oligomer binding and 4 replicates for monomer binding. NS  =  no significant binding above background.

**Figure 7:**
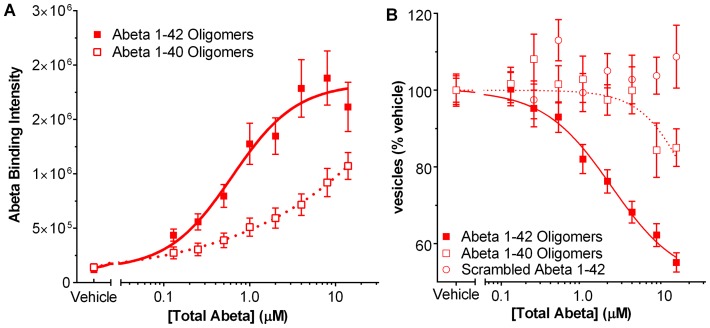
Effect of Abeta control peptides in membrane trafficking assay. Oligomer preparation of Abeta 1–40 is 200 times less potent than Abeta 1–42 in binding assay (**A**) and in membrane trafficking (**B**). Scrambled Abeta 1–42 is not active in the membrane trafficking assay and is not detectable by the antibody used for the Abeta binding assay.

To further demonstrate that the membrane trafficking and Abeta binding assays are specific for oligomers, we tested preparations of Abeta 1–40 and Abeta 1–42 with a scrambled amino acid sequence which were prepared by the same method as for 1–42 oligomers. In both the Abeta binding assay and the trafficking assay, Abeta 1–40 oligomer preps were less than 200-fold as potent as Abeta 1–42 (**Fig. 7A** **B**), reflecting the lower amount of oligomer produced with Abeta 1–40 as seen on Western gels (**Fig. 2A**). Scrambled Abeta 1–42 had no effect in the membrane trafficking assay (**Fig. 7B**). Scrambled peptide is not recognized by the antibody to Abeta 1–42 and thus its binding to neurons could not be directly measured.

Evidence from these binding experiments suggests that oligomers behave as a ligand, and bind to a single saturable receptor site on neuronal synaptic puncta. Because oligomer binding at this location is associated with the synaptotoxicity underlying Alzheimer's disease progression, and because oligomer-mediated toxicity has been shown to be reversible following oligomer washout [22], [60], we focused analysis of subsequent compound-mediated binding inhibition experiments specifically on oligomer binding at synaptic puncta, and used oligomer concentrations close to this Kd for those assay conditions.

### Screening for compounds that reverse and prevent trafficking defects caused by Abeta *in vitro*

**Figure 8:**
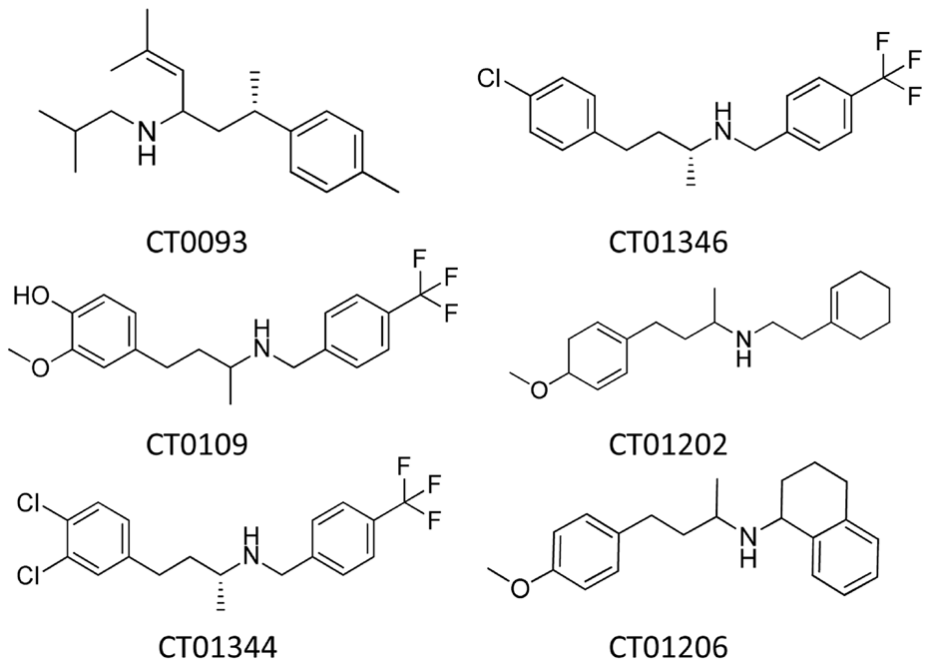
Chemical structures of compounds.

We screened a proprietary library of CNS drug-like small molecules to identify therapeutic candidates that block Abeta oligomer-induced vesicle trafficking deficits in mature, primary rat hippocampal and cortical cultures. Two novel, structurally distinct active molecules, CT0109 and CT0093, were identified as lead structures from which other active chemical analogs were synthesized. The structures of these compounds and of other chemical analogs of CT0109 – CT01344, CT01346, CT01202 and CT01206 are shown in **Fig. 8**.

**Figure 9:**
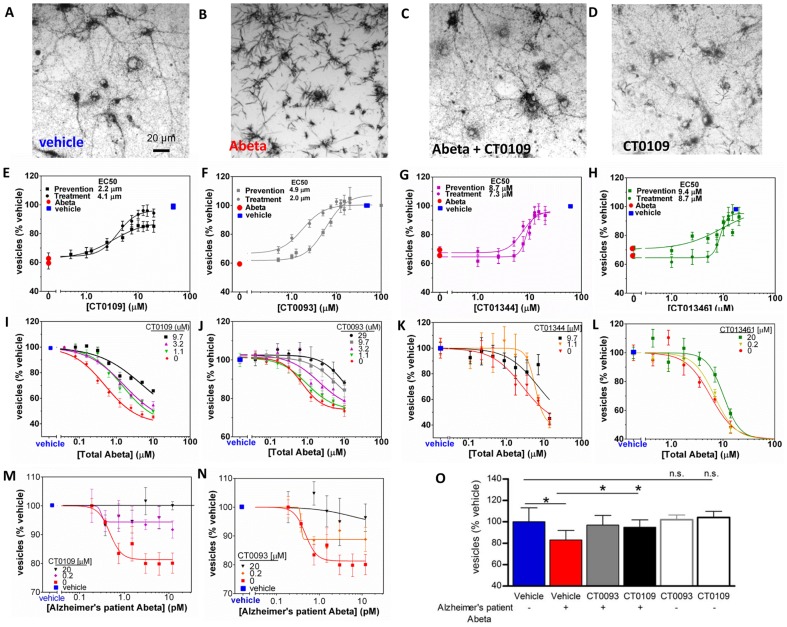
Abeta 1–42 oligomer-induced trafficking deficits are prevented and competitively inhibited by sigma-2/PGRMC1 antagonists. **A**, Reduced cargo dye (formazan) labels intracellular vesicles in vehicle-treated cultures, but is trafficked out of the cell more rapidly (**B**) following addition of synthetic Abeta oligomers (3 µM, total Abeta concentration). 15 µM CT0109 added 1 hr prior to Abeta restores trafficking to vehicle-treated levels (**C**) without affecting vesicles on its own (**D**). **E–H** Dose-response curves showing that CT0109 (**E**), CT0093 (**F**), CT01344 (**G**), and CT01346 (**H**) restore trafficking deficits whether added before (prevention) or after (treatment) addition of Abeta oligomers. **I–L**, synthetic Abeta oligomers exhibit a dose-dependent inhibition of membrane trafficking (red) that is right-shifted 5 to10-fold in the presence of increasing concentrations of sigma-2/PGRMC1 antagonists (**I**, CT0109: EC_50_ = 0.5 µM to 5.3 µM; **J**, CT0093: EC_50_ = 0.81 µM to 12.8 µM; **K**,CT01344: EC_50_ = 1.7 µM to 7.1 µM; **L**, CT01346 EC_50_ = 5.7 µM to 12.2 µM), consistent with pharmacological competition between compounds and Abeta oligomers. **M, N**, Abeta oligomers isolated from human postmortem AD patients exhibits a more potent dose-dependent inhibition of membrane trafficking (red), yet the same compounds inhibit the maximum effect of oligomers by 60 to 99% (P<0.025, t-test) making these oligomers less efficacious and therefore less toxic. **O**, CT0109 and CT0093 (0.2 µM, grey and black filled bars) reverse the trafficking deficit cause by human patient derived Abeta (1.5 pM, red bar) without affecting trafficking on their own (gray and black open bars).

CT0093, CT0109, CT01344 and CT01346 block the oligomer-induced trafficking deficits in a dose-dependent manner (**Fig. 9A–H** **,** **Table 4**). These compounds were effective when added either 1 hour prior to or 1 hour after Abeta oligomer preparations (**Fig. 9E–H**) showing that they can both prevent and reverse the effects of Abeta oligomers. When dosed against synthetic oligomers, ascending concentrations of CT0109, CT0093, CT01344 and CT01346 cause a progressive rightward shift in Abeta oligomer EC_50_ (**Fig. 9I–L**, Schild slope  = 1), indicating pharmacological competition with oligomers for access to targets mediating membrane trafficking. CT0109 and CT0093 also cause a dose-dependent shift in the dose-response effect of human AD patient-derived Abeta on membrane trafficking (**Fig. 9M, N**) without affecting trafficking on their own (**Fig. 9O**).

**Table 4:** Potency of compounds in membrane trafficking assay.

Compound ID	CT0093	CT0109	CT01344	CT01346	CT01202	CT01206
**Prevention**						
EC_50_ (µM)	4.9	3.2	8.7	9.4	6.1	4.3
Log EC_50_ ± SE	−5.31±0.05	−5.49±0.9	−5.06±0.02	−5.03±0.99	−5.12±0.38	−5.36±0.05
Goodness of fit (r^2^)	0.999	0.999	0.999	0.998	0.998	0.999
N	33	23	9	9	19	16
**Treatment**						
EC_50_ (µM)	2.0	4.1	7.3	6.4	n.d.	n.d.
Log EC_50_ ± SE	−5.70±0.13	−5.39±0.04	−5.19±0.11	−5.06±0.02	n.d.	n.d.
Goodness of fit (r^2^)	0.999	0.998	0.998	0.999	n.d.	n.d.
N	16	11	6	9	n.d.	n.d.

Prevention: compound added 1 hr prior to Abeta oligomers. Treatment: compound added 1 hr after Abeta oligomers. N =  number of experimental repeats (four replicate wells per experiment). n.d.  =  not determined.

### Anti-Abeta compounds prevent and reverse binding of Abeta to neurons, *in vitro*

**Figure 10:**
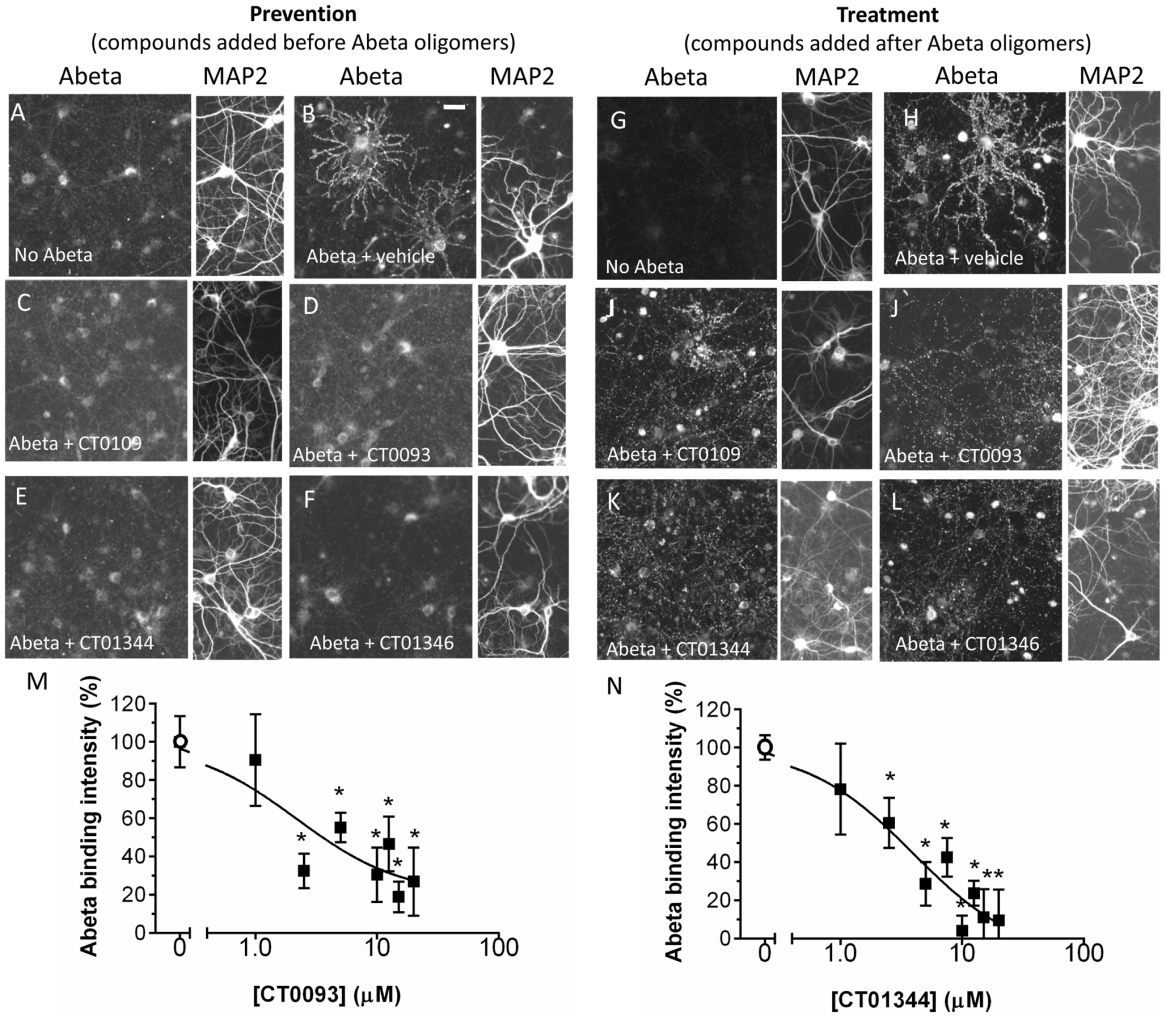
Small molecule therapeutic candidates can prevent or displace Abeta oligomer binding to mature primary hippocampal and cortical cultures (21DIV). Abeta synthetic oligomers were added to neuronal cultures (0.5 µM total Abeta concentration) for 40 min prior to (**A–F**) or following (**G–L**) addition of compounds and bound Abeta was detected by immunofluorescence. **A, G** Vehicle controls with no Abeta show background fluorescence. **C, I**, 15 µM CT0109, **D, J**, CT0093, **E, K**, CT01344, or **F, L**, CT01346 show that immuno-fluorescence for punctate binding of Abeta oligomers to neurites is blocked by all four compounds. Accompanying MAP2 panel shows similar density of neurons from right-hand side for each Abeta image. Scale bar  = 20 µm. **M**, Quantification of Abeta immunofluorescence shows that pretreatment with CT0093 prevents Abeta binding in a dose-dependent manner fitting a single site binding model (r2 = 0.79, N = 8, EC50  = 2.2±2.4 µM). **N**, CT01344 displaces pre-bound Abeta binding in a dose-dependent manner fitting a single site binding model (r2 = 0.92, N = 8, EC50  = 3.9±1.8 µM). *  =  statistically different than control, P<0.05, Student's t-test.

**Figure 11:**
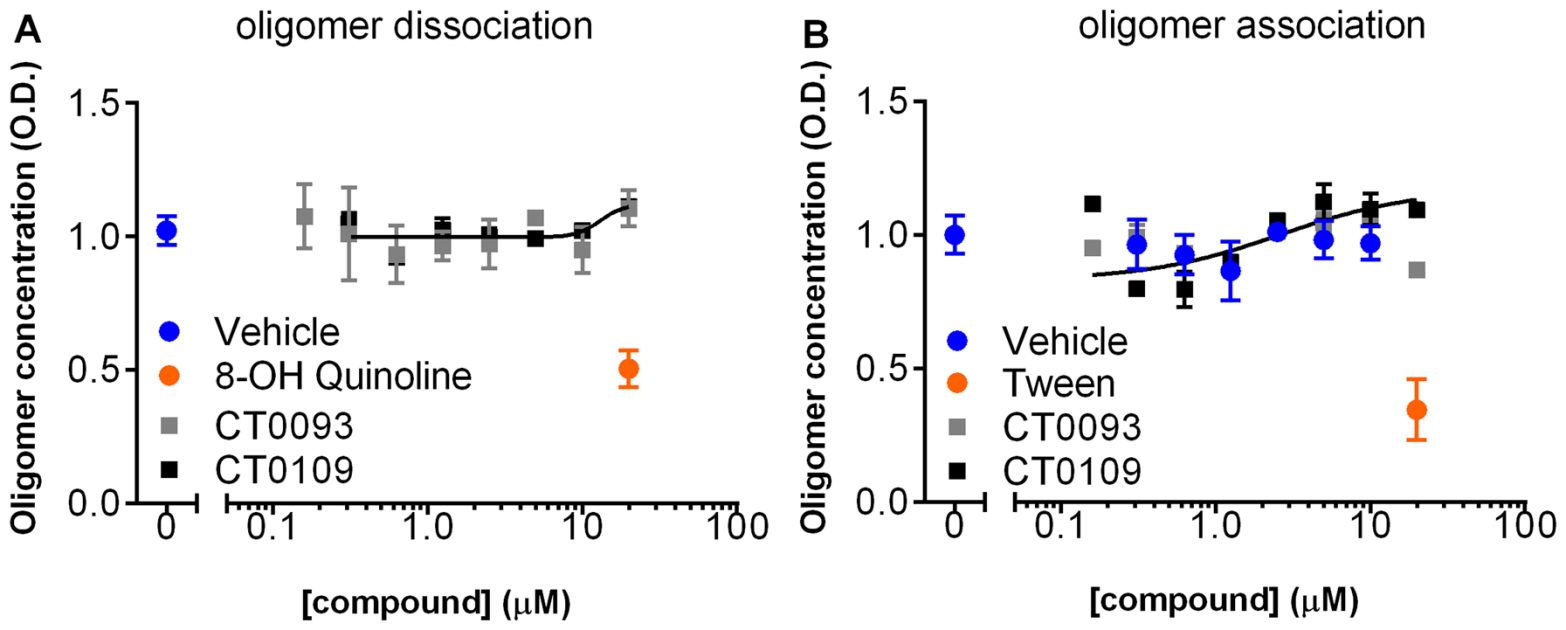
Small molecule Abeta binding antagonists do not act directly on Abeta oligomers. An ELISA specific for oligomeric forms of Abeta 1–42 shows that (A) preformed oligomers are dissociated by 8-OH quinoline but not by CT0109 or CT0093, and; (B) assembly of oligomers are inhibited by Tween but not by CT0109 or CT0093 at concentration of up to 20 µM for 24 hr, ruling out a direct effect of these compounds on oligomer assembly or disruption.

We examined whether these compounds could reduce Abeta oligomer binding to hippocampal/cortical cultures *in vitro*. Concentrations in this study represent the maximum dose of the compound in the membrane trafficking assay (**Fig. 9 E–H**) and an Abeta concentration equal to the Ki in the binding assay (**Fig. 6**). The addition of 15 µM CT0109, CT0093, CT01344 and CT01346 one hour prior to addition of synthetic Abeta oligomers (0.5 µM total Abeta concentration) all prevented the binding of Abeta oligomers to neuronal cultures by 99%±7%, 93%±2%, 94%±4% and 93%±4%, respectively (**Fig. 10A–F**). When the same concentration of these compounds were added 1 hour after addition of oligomers, all four compounds significantly displaced Abeta oligomers within 1 hour by 44%±3%, 49±5%, 66±14% and 93±13%, respectively (**Fig. 10G–L**). These compounds displace Abeta in a concentration dependent manner as shown for CT0093 in prevention (IC_50_ = 2.2 µM, **Fig. 10M**) and for CT01344 in treatment (IC_50_ = 3.9 µM, **Fig. 10N**). Compounds did not reduce the intensity of prebound Abeta oligomers as completely as they did when added to cultures before the oligomers. These displacement studies were performed after 1 hour treatment and it is possible that longer treatment with compounds can achieve a greater level of displacement. However, incubation with Abeta oligomers for longer times results in internalization of a portion of the Abeta oligomer labeling (27±8% internalization at 2 hr, **Fig. S2**), complicating the quantification of the compound treatment effects. In separate biochemical assays using an oligomer-specific ELISA, these small molecules do not appear to directly interact with oligomers and do not disrupt preformed oligomers or block formation of oligomers (**Fig. 11**). Since glial cell body binding at the oligomer concentrations used in these compound binding prevention and displacement experiments is so low (<10% of puncta binding intensity), we did not analyze the compound's ability to displace Abeta binding.

These results demonstrate that these compounds, discovered by screening for activity in the trafficking assay, are dose-dependent antagonists of Abeta oligomer binding. This is the first demonstration of dose-dependent displacement of Abeta oligomers by a small molecule.

### Anti-Abeta compounds prevent Abeta-induced synapse loss *in vitro*

**Figure 12:**
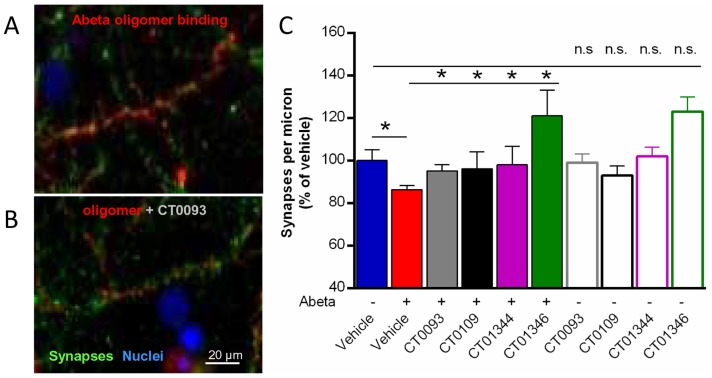
Small molecule Abeta binding antagonists prevent Abeta 1–42 oligomer-induced synaptic regression in cultured neurons. **A**, Abeta oligomers bound to a subset of neurites (red) reduces synaptophysin-immunoreactive synaptic puncta (green). **B**, Treatment with sigma-2/PGRMC1 antagonists reduces oligomer binding and restores normal immunoreactivity for the synaptic marker. **C**, Oligomers induce an average 18%±2 s.e.m. loss in the number of immunoreactive puncta per micron length of neurite (red bar) compared to vehicle-treated cultures (blue bar). Treatment of cultures with sigma-2/PGRMC1 antagonists (closed bars) restores synaptophysin immunoreactivity to normal, but has no effect when antagonists are dosed alone (open bars). *p = 0.05, Student's paired t-test.

In addition to altering rates of membrane trafficking, oligomers have been shown to induce reversible spine retraction *in vitro* [22] and to cause a corresponding loss of synapses and synaptic proteins including synaptophysin [21], [47]. Addition of synthetic Abeta oligomers to neuronal cultures caused an 18% loss of synaptophysin-immunoreactive puncta *in vitro* (**Fig. 12A,C**) compared to vehicle treatment; this is similar to the degree of synapse loss seen using ultrastructural stereology methods in post-mortem hippocampus from humans diagnosed with Mild Cognitive Impairment [61]. Small molecule anti-Abeta compounds eliminate oligomer-induced loss of synaptophysin-positive puncta (**Fig. 12B, C**), with no effect on puncta number when dosed alone.

### Anti-Abeta compounds are effective *in vivo*

**Figure 13:**
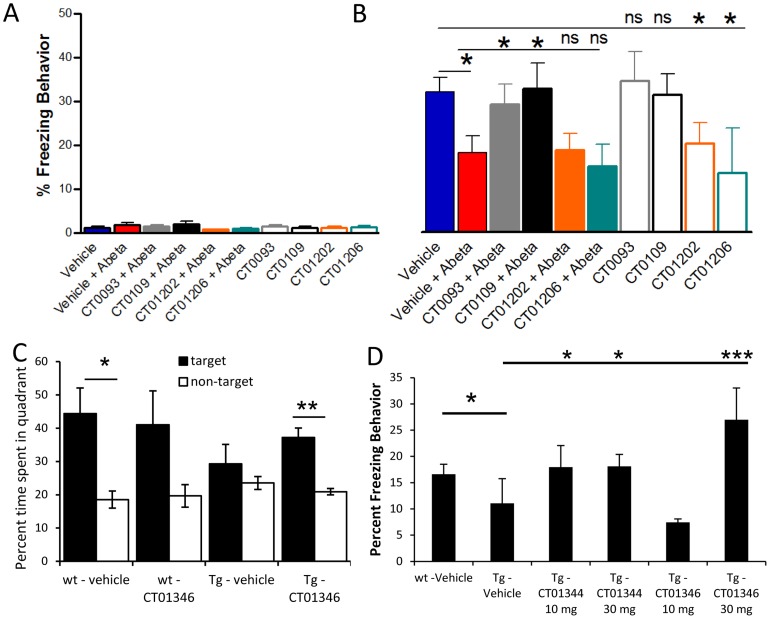
Small molecule Abeta binding antagonists improve cognitive deficits in mice. **A,B**, sigma-2/PGRMC1 antagonists prevent oligomer-induced contextual fear conditioning memory deficits in C57BL/6 male mice. **A**. No behavioral deficits are observed during fear conditioning training with any treatment. **B**. Testing 24 hours after training reveals that a single injection (2 µM) of Abeta antagonists CT0093 (solid gray bar) or CT0109 (solid black bar) via bilateral intrahippocampal injection one hour prior to oligomer injection (200 nM) prevents oligomer-induced fear memory deficits (solid red bar;CT0109: *p = 0.03, CT0093: *p = 0.05, pairwise t-test comparing Abeta vs. Abeta plus compound). Treatment with compound in the absence of Abeta oligomers does not result in fear memory deficits (open grey and black bars, N = 10–18 animals/group). Treatment with CT01202 or CT01206 (2 µM) did not prevent Abeta oligomer-induced memory deficits (solid orange and green bars, ns  =  not significant by paired t-test comparing Abeta vs. Abeta plus compound, N = 12, 9, respectively) and caused fear memory deficits in the absence of Abeta (open orange and green bars, *p = 0.05, paired t-test, vehicle, vs compound alone, N = 11, 8 respectively). **C**. Abeta oligomer antagonists rapidly improve cognitive deficits in aged transgenic mice. Eleven month old female hAPP Swe/Ldn transgenic or wild-type littermates treated for 42 days with CT01346 at 30 mg/kg/day p.o. significantly improves transgenic animal spatial memory retrieval performance in Morris water maze probe trial (**p = 0.005, paired t-test, N = 7–9 animals/group). **D**. Abeta oligomer antagonists sustain cognitive improvement in aged transgenic mice. Nine month old male hAPP Swe/Ldn transgenic mice treated for 5.5 months with vehicle or Abeta antagonists CT01344 at 10 and 30 mg/kg/day or CT01346 at 30 mg/kg/day p.o. significantly improves transgenic animal contextual fear conditioning memory deficits (*p = 0.0237,*p = 0.25, ***p = 0.0005, respectively, Mann Whitney U test, N = 13–15 animals/group).

The compounds that were found in our *in vitro* screens are all brain penetrant, reaching appreciable concentration in the brain as evidenced by measurements made 24 hours after dosing in mice or rats (**Table 5**). Oligomers cause memory loss when administered acutely to rodents [26], [62] or as a result of age-dependent oligomer accumulation in transgenic animals [31], [63]. Compounds did not affect motor activity measured following acute (**Fig. 13A**, contextual fear conditioning task training baseline) or long-term administration (Morris water maze swim speed, data not shown). Abeta oligomers cause a deficit in contextual fear conditioning-dependent associative memory when injected directly into the dorsal hippocampi of wild-type, male C57/BL6 mice 24 hours prior to testing [62] (**Fig. 13B**, red bar, p = 0.01, pairwise Student's t-test Abeta vs. vehicle, blue bar). CT0109 and CT0093 (2 µM) injected one hour prior to oligomer injection completely prevented fear memory deficits (**Fig. 13B**, filled black and gray bars, CT0109 p = 0.03, CT0093, p = 0.05, Student's t-test comparing Abeta vs. Abeta + compound conditions), but have no effect on memory when administered alone (**Fig. 13B**, open black and gray bars). Two additional compounds which were active in the membrane trafficking assay, CT01202 and CT01206 (**Fig. 8**, **Table 4**), were not effective in preventing memory deficits caused by Abeta oligomers (**Fig. 13B**, filled orange and green bars). These compounds were neuroactive, causing memory loss when injected alone without Abeta (**Fig. 13B**, open orange and green bars). These compounds also affect synapse number when dosed in the absence of Abeta oligomers *in vitro* (**Table S1**).

**Table 5:** Correlation of brain concentration of compounds with behavioral efficacy and estimated receptor occupancy at sigma-2/PGRMC1 receptor.

Compound (Ki, nM)	CT0093	(54)	CT0109	(9)	CT01344	(48)	CT01346	(50)
Dose (mg/kg/day)	1	10	1	10	10	30	10	30
Compound Concentration in brain (nM)	64	555	41	266	394	793	54	330
Measured efficacy	-	+	+	+	+	+	-	+
Estimated % receptor occupancy	54%	**91%**	**82%**	**97%**	**48%**	**94%**	52%	**87%**

CT0093 and CT0109 were dosed subcutaneously in mice by continuous osmotic minipumps infusions at the doses indicated. CT01344 and CT01346 were dosed by once daily oral gavage. Twenty four hours after the last dose, animals were euthanized and drug concentration in the brain was measured. Ki  =  binding affinity of compound at the sigma-2/PGRMC1 receptor. Measured efficacy: statistically significant improvement (+), or no significant improvement (-) seen in behavioral tests. Estimated % receptor occupancy was calculated according to the formula (concentration/Ki)/[(concentration/Ki) + 1)], where Ki is determine by radioligand competition binding.

Transgenic mice overexpressing the human APP gene with Swedish and London familial mutations accumulate Abeta in the brain with age [64] and exhibit age-dependent learning and memory deficits [37]. Eleven month-old female transgenic mice exhibit deficits in spatial memory as measured by the Morris water maze probe trial test. Non-transgenic vehicle-treated animals spend significantly more time in the target quadrant compared to transgenic vehicle-treated animals (**Fig. 13C**, p = 0.036, pairwise Student's t-test). Oral administration of 30 mg/kg/day of CT01346 to transgenic animals for 42 days significantly improves spatial memory retrieval performance (p = 0.005 paired Student's t-test vs. transgenic vehicle-treated group). Nontransgenic animals treated with CT01346 behaved similarly to vehicle-treated wild-type animals (**Fig. 13C**).

In order to determine if the compound-mediated improvement in cognitive performance in transgenic animals is sustained, transgenic 9 month old male mice treated with vehicle, 10 or 30 mg/kg/day of CT01344 or CT01346 for 5.5 months p.o., as well as non-transgenic vehicle-treated littermates were tested for contextual fear conditioning memory formation (**Fig. 13D**). When the animals were tested for contextual fear memory 24 hours after training, transgenic mice performed significantly worse compared with the non-transgenic vehicle-treated animals (Mann-Whitney U test, p = 0.0246). Transgenic animals treated with 10 and 30 mg/kg/day of CT01344 (p = 0.0237, p = 0.025, respectively) and 30 mg/kg/day of CT01346 (p = 0.0005) exhibited significantly improved fear memory performance compared to vehicle-treated transgenic animals (Mann-Whitney U test, **Fig. 13D**). Similar weight gain and mortality in treated and control groups reflect the specific effects of the compound on Abeta-mediated behavioral deficits. We conclude that these anti-Abeta antagonists are capable of preventing and reversing established memory deficits in both sexes in aged transgenic AD mouse models following systemic long-term administration, and represent therapeutic disease-modifying candidates for Alzheimer's disease.

We counter-screened these behaviorally effective molecules in a panel of 100 targets present in the brain, including major receptors, ion channels and enzymes which could affect synaptic plasticity and found that the efficacious small molecules compete selectively with high affinity for radioligand binding to sigma-2/PGRMC1 receptors [65]. We also measure the brain concentrations of the test compounds in the mouse models. Examination of the affinities of the compounds indicates that the measured brain concentrations at doses that restored memory to normal following chronic administration in transgenic Alzheimer's mouse models corresponds to a greater than 80% receptor occupancy (%RO) at sigma-2/PGRMC1 receptors (**Table 5**). Brain concentrations corresponding to 50% receptor occupancy were not effective at restoring memory, suggesting that the sigma-2/PGRMC1 is the target for these compounds.

## Discussion

This manuscript describes a series of assays for measuring the effects of multiple preparations of Abeta oligomers *in vitro*, and the use of those assays to find small molecule antagonists of Abeta oligomers that are capable of reversing cognitive defects in mouse models of Alzheimer's disease. In primary cultures of rat hippocampal and cortical neurons 21DIV, these compounds prevent and displace the binding of Abeta oligomers to neuronal receptors, prevent and reverse the effects of Abeta oligomers on membrane trafficking and prevent the loss of synapses caused by Abeta. Activity of compounds in these *in vitro* assays was predictive for behavioral efficacy *in vivo* (**Table S1**).

The current studies provide evidence that synthetic and human-derived Abeta oligomers act as pharmacologically-behaved ligands at neuronal receptors, i.e. they exhibit saturable specific binding to a target, they exert a functional effect related to their binding and their displacement by small molecule antagonists blocks their functional effect. The first-in-class small molecule receptor antagonists described here restore memory to normal in multiple AD models and sustain improvement long-term. These compounds represent a novel mechanism of action for disease-modifying Alzheimer's therapeutics.

Evidence suggests that Abeta oligomers reduce neuronal surface receptor expression through changes in membrane trafficking. These changes are the basis for oligomer inhibition of electrophysiological measures of synaptic plasticity (LTP) and thus learning and memory [20], [66]. Measuring changes in membrane trafficking rate induced by oligomers using morphological shifts in formazan has been used in cell lines to discover Abeta oligomer-blocking drugs [51], [52], [67]–[69] which lower Abeta brain levels in rodents *in vivo* [70]. Liu and Schubert [51] discovered that cells respond to sublethal concentrations of Abeta oligomers by selectively accelerating the exocytosis rate of reduced formazan, while leaving endocytosis rate unaffected. We and others have found that this assay is sensitive to low levels of oligomers that do not cause cell death [52], [68]. The dual applicability of this assay to measure both cell death and membrane trafficking rate under different conditions has led to some confusion in the literature [71], particularly since low amounts of oligomers that lead to inhibition of LTP do not lead to cell death [72]. All of our assays are conducted with non-lethal concentrations of Abeta oligomers.

There are large differences in the potency of synthetic Abeta oligomers and human derived Abeta in the trafficking assay, perhaps due to the presence of large amounts of monomer in synthetic preps. However, we have found that the ability of compounds to block the effects of multiple sources of Abeta oligomers without having effects on their own is predictive for their ability to restore cognitive function in *in vivo* models of Alzheimer's disease caused by age-related increases in Abeta (**Table S1**). Thus, although our knowledge of the exact pathological species of Abeta oligomer is incomplete, by using multiple assays and multiple preps we have discovered disease-modifying therapeutic candidates with the potential to modify the course of the disease.

We have linked this membrane trafficking assay with Abeta binding and synapse counting assays into a platform for discovering anti-Abeta drugs which restore cognitive ability *in vivo*. The binding of synthetic Abeta oligomers to puncta on neuronal neurites shows concordance with their functional effect in altering trafficking rates, with a binding affinity (436±67 nM, **Fig. 5**) agreeing with an EC_50_ in the functional assay (462±56 nM, **Fig. 6**). The concentration of compounds needed to inhibit binding of Abeta oligomers is in good agreement with the concentration which inhibits the effects of Abeta oligomers on membrane trafficking and synapse loss *in vitro*. For example, CT01344 blocked the effects of Abeta on membrane trafficking with an EC_50_ of 8.7±0.4 µM (**Fig. 9**) and displaced Abeta binding on cultured neurons with an EC_50_ of 3.9±1.8 µM (**Fig. 10**). CT01344 restored synapse loss induced by Abeta 100% at 15 µM (**Fig. 12**) The low dynamic range of synapse loss seen with the non-lethal concentrations of synthetic oligomers used in all our experiments makes it difficult to observe a dose-dependent effect. The concentration of compound used is at the top of the dose-response curve for CT01344 inhibition of oligomer-induced membrane trafficking deficits (**Fig. 9**). Thus, there is a good correlation between potency of the synthetic Abeta oligomer preparation in the different *in vitro* assays as well as a good correlation with potency of the compounds in those assays.

In behavioral studies in transgenic mice, all four of the compounds we have studied must reach a brain concentration that exceeds a theoretical receptor occupancy at sigma-2/PGRMC1 of greater than 80% to be behaviorally effective (**Table 5**). The EC_50_ for the effects of CT01344 *in vitro* is nearly two orders of magnitude above its binding affinity for sigma-2/PGRMC1 as determined by radioligand binding studies (48±5 nM). This offset between affinity at sigma-2/PGRMC1 could be due to one or more factors: 1) physico-chemical interactions of the compound such as binding to microtiter plate plastic, could lower the effective concentration of the compound in *in vitro* assays, 2) greater than 95% of sigma-2/PGRMC1 receptors need to be occupied by compound to achieve an effect on binding and function, or 3) the high concentration of the low potency synthetic Abeta needed to achieve adequate testing windows in the *in vitro* assays. The first possibility likely plays some role, due to the relative lipophilicity of these compounds – a property which is desired for compounds to penetrate the blood-brain barrier. The second possibility indicates that the binding site of the compounds is not identical to the binding site of Abeta oligomers but rather, that the compounds act to alter the affinity or surface expression of the Abeta oligomer binding site and that multiple drug binding sites must be occupied to block the binding of Abeta oligomers (i.e., the pharmacological concept of “spare receptors”). The third explanation (that a vast excess of compound is needed to overcome a vast excess of Abeta oligomers) would be consistent with a direct competition between the compounds and Abeta, however, this is unlikely given the compound's ability to displace already-bound oligomers (**Fig. 10**) under non-equilibrium conditions. This latter evidence suggests that compound is modulating the receptor site allosterically to increase Abeta oligomer off-rate.

It is challenging to construct a model of how our compounds might work to block Abeta oligomer binding and effects in the absence of precise information about the structure of the Abeta oligomer ligand. Recent reviews have accurately pointed out that we lack a complete understanding of the precise structure of the oligomer ligand [32], [73], and it would appear difficult to conduct drug discovery studies in the absence of this information. This paper lays out a roadmap by which this may be possible: by systematically comparing the effects of different kinds of oligomers on the same assays. In this context, the effective small molecules themselves become tools that reveal downstream biology common to a wide range of oligomers (some of which may exist in the brain as equilibrium mixtures of structures).

The non-biased, phenotypic screens we used for finding compounds that inhibit the effects of Abeta oligomers in cultured neurons could have detected compounds working by several different mechanisms of action, including disruption of oligomers themselves (oligomer dissociation), interference with Abeta oligomer binding to the surface of neurons, or interference with signaling mechanisms downstream from the binding of Abeta oligomers. We have ruled out the direct dissociation of oligomers or the inhibition of oligomer formation (**Fig. 11**) and have identified our active compounds as antagonists of Abeta oligomer binding.

There are different models which could explain the prevention and displacement of Abeta binding by small molecules. The small molecules could bind directly to Abeta receptor protein and compete for binding at the same site or at a distal site on the same protein, or the compounds could bind to a protein that is a modulator of the Abeta receptor protein, affecting the affinity of that receptor protein for Abeta oligomers.

We observed a progressive rightward shift in the dose-response curve to synthetic and human-derived Abeta oligomers in the membrane trafficking assay in the presence of increasing concentrations of test compounds, suggesting competitive antagonism of Abeta oligomers (**Fig. 9I–L**). However, the trafficking assay is a functional assay downstream of oligomer binding, in which allosteric interaction with a binding site for Abeta could appear to be a competitive interaction. The competition of compounds versus human derived Abeta appears to affect the magnitude of the response in addition to the potency (**Fig. 9M, N**) of the Abeta. This effect could be a result of a real difference in coupling efficiency between the human vs. synthetic oligomers and their receptor targets. Alternatively, this may be an artifact due to truncation of the top of the dose response curve due to insufficiently high concentrations of human Abeta. The present data cannot distinguish between these two possibilities. We observed both inhibition of binding of Abeta oligomers and displacement of prebound synthetic Abeta oligomers. The latter effect was not as complete as the former, which could be a function of the short time of incubation (1 hr). Examining longer time points for displacement of Abeta oligomers is problematic in living cells because a fraction of the Abeta is internalized with longer incubations (27%±8% internalized after 2 hour, **Fig. S1**). With long-term treatment (such as was done in the behavioral experiments, *in vivo*), the presence of compound likely prevents the binding of additional Abeta oligomers over a period of time when any Abeta initially bound has been internalized and degraded by cells, thus providing long-term effectiveness of the compounds.

Thus, the present data is consistent either with a model in which these compounds are binding directly to the receptor for Abeta oligomers or with a model in which the target for these compounds is acting to modulate the receptor for Abeta oligomers. Many candidate oligomer receptors have been proposed [7]–[9], [11]–[13], [15], but the identity of these putative receptors have not been accompanied by the discovery of small molecule ligands with the ability to displace oligomers from their receptors. We have identified the sigma-2/PGRMC-1 receptor as the molecular target of these compounds. Further evidence for the role of sigma-2/PGRMC1 in mediating the binding and effects of Abeta oligomers is the subject of a separate paper [65].

Regardless of exact mechanism, the present data demonstrate that Abeta oligomers are specific ligands for cell surface receptors at synapses showing defined single-site saturable binding that is associated with down-stream functional effects. We have also demonstrated that small molecules can be used to antagonize the binding of Abeta oligomers and to inhibit the down-stream effects of those oligomers on membrane trafficking and synapse loss. These same molecules are effective at preventing and reversing cognitive deficits in two animal models for Alzheimer's disease in both genders, following short or long systemic administration indicating that tolerance to these compounds does not readily develop. These molecules are the first to be identified as functional antagonists of Abeta oligomer binding on neuronal cells.

The current studies did not examine the effects of compounds on Abeta load or accumulation in plaques as it is unlikely that compounds that block binding of Abeta oligomers will affect those parameters. A recent study using a TET-off APP overexpressing mouse model observed cognitive improvement in the absence of changes in amyloid plaques when the transgenic APP gene was turned off [74]. This finding supports the hypothesis that blocking the binding and effects of Abeta oligomers can have a therapeutic effect in the absence of plaque clearance.

Synaptic plasticity processes underlying learning and memory are complex, and many receptors participate in a brain circuit location- and state-dependent manner. Oligomers have been demonstrated to interact with signaling pathways downstream from a number of receptors.

AD is one of many proteopathy-related diseases including Huntington's disease, Parkinson's and prion diseases. This report is the first demonstration that a pathogenic protein assembly, Abeta 1–42 oligomers, acts as a pharmacologically behaved ligand at a receptor site on target brain cells and are therapeutically tractable to inhibition by small molecule antagonists. Oligomers display saturable binding to localized sites on neurons, and their binding and downstream synaptotoxic effects can be prevented and competitively displaced by small molecule therapeutics. This pharmacological mechanism ensures that these therapeutics will continue to be effective disease-modifying agents as concentrations of oligomers rise throughout the disease process. These first-in-class, highly brain-penetrant, disease-modifying amyloid oligomer receptor antagonists restore memory to normal in multiple AD models and sustain improvement long-term. This demonstration, that there is a pathological protein ligand-receptor interaction underlying Alzheimer's disease, and that it can be stopped with small molecule pharmacology, is good news for patients, for whom no disease-modifying therapies currently exist.

## Supporting Information

Figure S1 **Differentiation of glial population by nuclear morphology.** The morphology of MAP-2 negative glial nuclei labeled with DAPI was analyzed via automated image processing. **A.** Nuclear labeling with DAPI for each glial cell is graphed as (**A**) total intensity versus average intensity and as (**B**) variable intensity versus area. Two broad populations of glial cells are seen: One population with a round, symmetrical nuclear morphology does not bind Abeta oligomer or monomer (red circle in **A**, **B**, and images in **C**), and a second population (black dotted lines in **A** and **B**) characterized by a condensed (blue circle in **A**, **B**, and images in **D**) or asymmetrical nuclear morphology (green circle in **A**, **B** and images in **E**) binds Abeta oligomer and monomer on its cell body with equal brightness. Data from glia in these populations are analyzed separately for Abeta binding quantified in **Figure 6** and **Table 3**. Abeta images are from cells treated with 4 µM of synthetic oligomeric Abeta for 30 min.).
(TIF)

Figure S2 **Internalization of Abeta oligomers.** After treatment with Abeta oligomers (1 µM) for 1 hr (**A, B**) or 2 hr (**C, D**), cultures were fixed and immunolabeled for Abeta either in the presence of 0.05% Triton X-100 to permeabilize cells to immunoglobulins and measure all Abeta present (**A, C**) or in the absence of this detergent (**B, D**) to detect only Abeta bound at the cell surface. Control labeling with antibody for MAP2 shows that this intracellular protein is detectable only in the presence of detergent (**E**) and not in its absence (**F**). **G, H,** Quantification of total Abeta and surface Abeta shows that after 1 hr of exposure, 96%±8% S.E.M. of the bound Abeta was at the surface, while after 2 hrs, 73%±3% S.E.M. of the Abeta bound was at the surface, while 27%±8% S.E.M. of the Abeta was internalized.
(TIF)

Table S1 **Predictivity of** ***in vitro*** **assays for** ***in vivo*** **behavioral efficacy.**
(DOCX)
