# Supplementary figures and images for: Alzheimer's Therapeutics Targeting Amyloid Beta 1–42 Oligomers I: Abeta 42 Oligomer Binding to Specific Neuronal Receptors Is Displaced by Drug Candidates That Improve Cognitive Deficits
Source: PLoS One. 2014 Nov 12;9(11):e111898. doi: 10.1371/journal.pone.0111898 (PMC4229098; doi:10.1371/journal.pone.0111898)

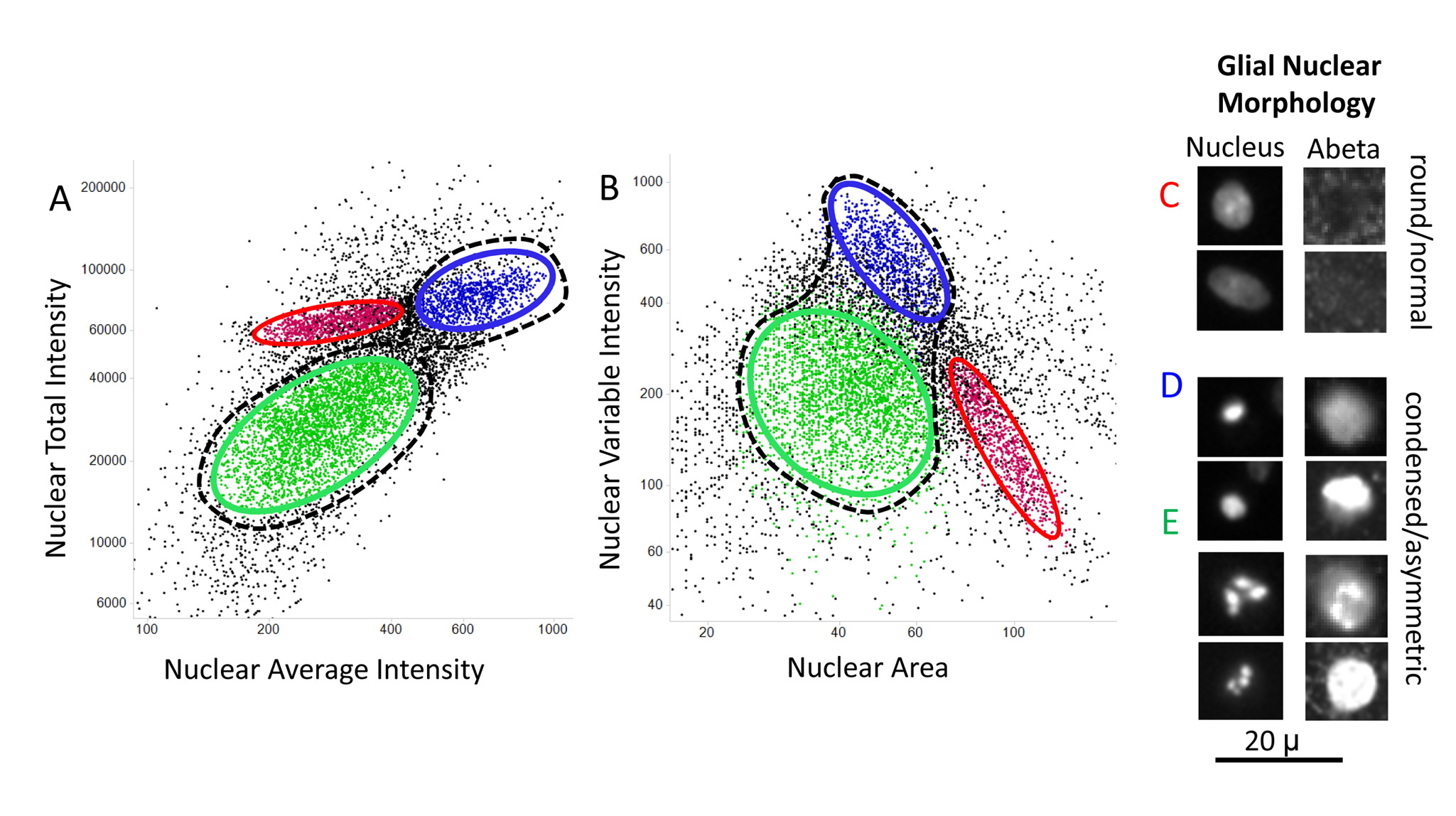

Supplement: Figure S1 — Differentiation of glial population by nuclear morphology. The morphology of MAP-2 negative glial nuclei labeled with DAPI was analyzed via automated image processing. A. Nuclear labeling with DAPI for each glial cell is graphed as (A) total intensity versus average intensity and as (B) variable intensity versus area. Two broad populations of glial cells are seen: One population with a round, symmetrical nuclear morphology does not bind Abeta oligomer or monomer (red circle in A, B, and images in C), and a second population (black dotted lines in A and B) characterized by a condensed (blue circle in A, B, and images in D) or asymmetrical nuclear morphology (green circle in A, B and images in E) binds Abeta oligomer and monomer on its cell body with equal brightness. Data from glia in these populations are analyzed separately for Abeta binding quantified in Figure 6 and Table 3 . Abeta images are from cells treated with 4 µM of synthetic oligomeric Abeta for 30 min.). (TIF) [file pone.0111898.s001.tif]

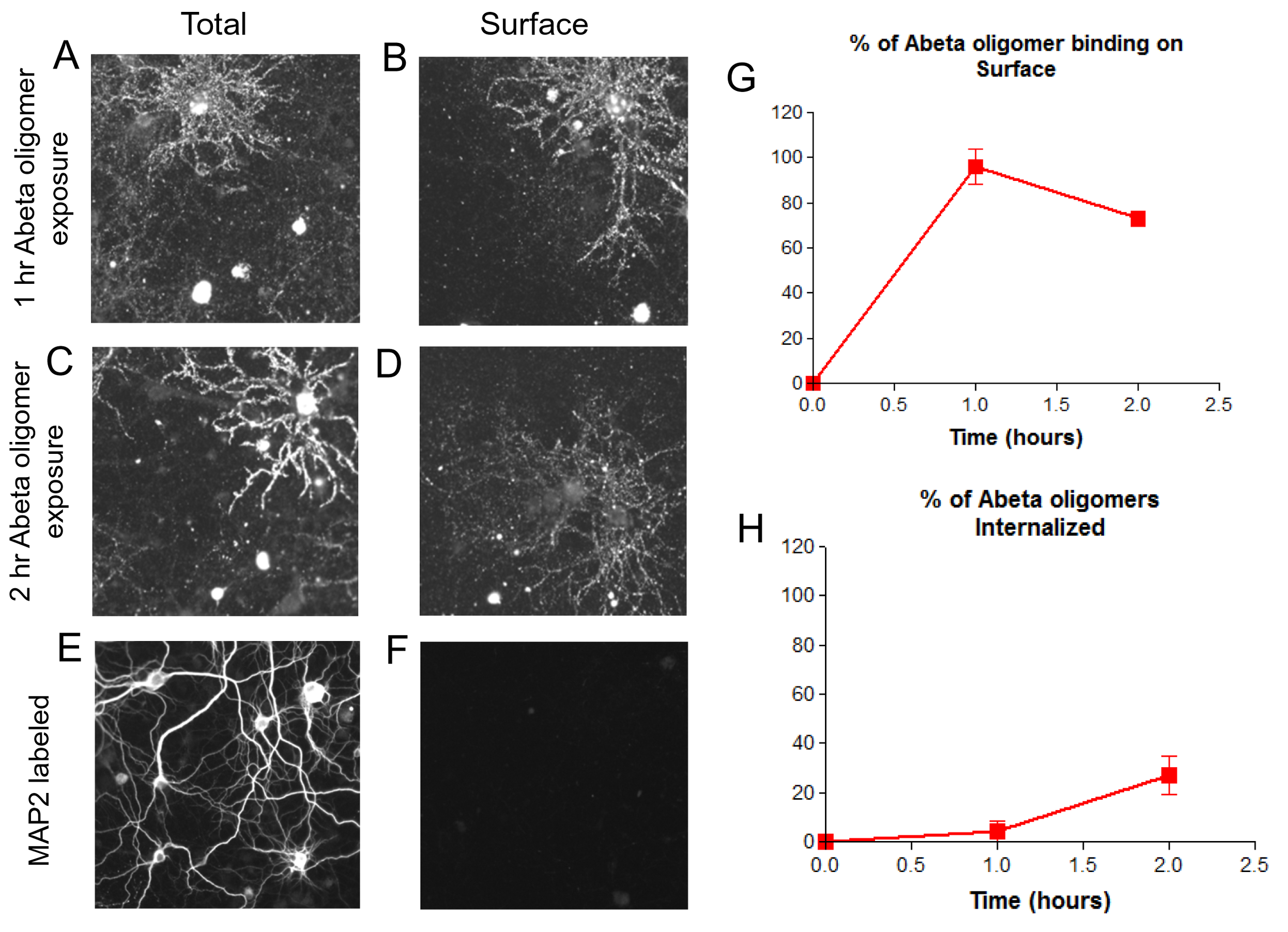

Supplement: Figure S2 — Internalization of Abeta oligomers. After treatment with Abeta oligomers (1 µM) for 1 hr (A, B) or 2 hr (C, D), cultures were fixed and immunolabeled for Abeta either in the presence of 0.05% Triton X-100 to permeabilize cells to immunoglobulins and measure all Abeta present (A, C) or in the absence of this detergent (B, D) to detect only Abeta bound at the cell surface. Control labeling with antibody for MAP2 shows that this intracellular protein is detectable only in the presence of detergent (E) and not in its absence (F). G, H, Quantification of total Abeta and surface Abeta shows that after 1 hr of exposure, 96%±8% S.E.M. of the bound Abeta was at the surface, while after 2 hrs, 73%±3% S.E.M. of the Abeta bound was at the surface, while 27%±8% S.E.M. of the Abeta was internalized. (TIF) [file pone.0111898.s002.tif]
