# Supplementary material for: Alzheimer's Therapeutics Targeting Amyloid Beta 1–42 Oligomers I: Abeta 42 Oligomer Binding to Specific Neuronal Receptors Is Displaced by Drug Candidates That Improve Cognitive Deficits
Source: PLoS One. 2014 Nov 12;9(11):e111898. doi: 10.1371/journal.pone.0111898 (PMC4229098; doi:10.1371/journal.pone.0111898)
Supplement: Table S1 — Predictivity of in vitro assays for in vivo behavioral efficacy. (DOCX) [file pone.0111898.s003.docx]

**Table S1: Predictivity of *in vitro* assays for *in vivo* behavioral efficacy.**

| **Compound** | **Block Aβ-induced membrane trafficking deficits EC50 (μM)** | **Block Aβ-induced synapse loss** | **Affects Synapse density in the absence of Aβ** | **Behavioral efficacy** |
| --- | --- | --- | --- | --- |
| CT0109 | 2.2 | +++ | No | Yes |
| CT0093 | 4.9 | +++ | No | Yes |
| CT01344 | 8.7 | +++ | No | Yes |
| CT01346 | 9.4 | +++ | No | Yes |
| CT01202 | 6.1 | +++ | Yes | No |
| CT01206 | 4.3 | +++ | Yes | No |
